# A direct test of the belongingness hypothesis: how fully vs. partially satisfying relationships relate to perceived belongingness and well-being

**DOI:** 10.3389/fpsyg.2026.1830244

**Published:** 2026-10-07

**Authors:** Glenn P. Malone, David R. Pillow, Meghan A. Crabtree, Janelle Kohler

**Affiliations:** 1 Department of Psychology, University of Texas, San Antonio, TX, United States; 2 Tri-Ethnic Center for Prevention Research, Colorado State University, Fort Collins, CO, United States

**Keywords:** belongingness hypothesis, diminishing returns, partial relationships, satisfying relationships, sense of belonging, well-being, whole relationships

## Abstract

**Introduction:**

Vital components of the “belongingness hypothesis” set forth by Baumeister and Leary were examined: (a) fully satisfying relationships—referred to here as “whole” relationships—are defined by a set of criteria, and some small number of these relationships are required to meet one’s belongingness needs; (b) relationships that do not meet these criteria do not predict belongingness and well-being as do whole relationships; and (c) the satisfaction of maintaining additional relationships beyond the minimum number is diminished. The present research examined whether the benefits of interpersonal relationships diminish as the number of whole relationships increases.

**Methods:**

Collegiate samples (*N* = 872, 581, and 284) listed their close relationships and answered questions about the nature and quality of those relationships with respect to theoretically specified criteria. Structural equation modeling was used to estimate the linear and quadratic associations between numbers of whole relationships and assessments of belongingness and well-being.

**Results and discussion:**

Fully satisfying relationships specifically classified by a set of criteria were positively related to belongingness and well-being. In contrast, increasing numbers of interpersonal relationships that failed the criteria for a whole relationship were not positively related to belongingness, but instead were negatively related. Finally, although whole relationships showed linear associations with belongingness and subjective well-being, significant quadratic effects were also found in two of three studies. That is, gains in belongingness and well-being showed evidence of gradually diminishing as the number of whole relationships extended beyond minimum requirements. The results support the utility of distinguishing between whole and partial relationships as originally theorized.

## Introduction

In their 1995 seminal review, Baumeister and Leary proposed that the motive to belong represents a fundamental human need, one that if met, is expected to strongly predict subjective well-being. The literature on belongingness has grown immensely in the 30 years since this review. Based on a sampling of this large literature, it has been established that belongingness relates positively to well-being (e.g., Arslan and Allen, 2021; Michalski et al., 2020) and negatively to pathology (e.g., Choenarom et al., 2005; Kissan and McClaren, 2006).

Surprisingly, little research has directly assessed key elements of the Baumeister and Leary (1995) belongingness hypothesis pertaining to specific criteria for defining the nature of one’s interpersonal relationships. These criteria are evident in the following statement: “The belongingness hypothesis is that human beings have a pervasive drive to form and maintain at least a minimum quantity of lasting, positive, and significant interpersonal relationships. Satisfying this drive involves two criteria: First, there is a need for frequent, affectively pleasant interactions with a few people, and, second, these interactions must take place in the context of a temporally stable and enduring framework of affective concern for each other’s welfare” (Baumeister and Leary, 1995, pg. 497).

To fully satisfy one’s belongingness needs, Baumeister and Leary (1995) claimed that people need more than just frequent positive interactions with various others; they also need meaningful interactions with the same person(s) over time. Specifically, they argued that people require a sense of stability and continuity in their relationships. This notion, that humans need interpersonal relatedness characterized by both regular contact and an ongoing bond, is consistent with early research by Weiss (1973) and Shaver and Buhrmester (1983). These authors suggested that feelings of loneliness can be precipitated either by an insufficient amount of social contact (social loneliness) or by a lack of meaningful, intimate relatedness (emotional loneliness). From an evolutionary perspective, Baumeister and Leary (1995) theorized that relationships characterized by both frequent contact and meaningful connectedness would have greater survival and reproductive value than relationships characterized by only one of these features.

There is a plethora of studies that have examined portions of the above proposition. For example, Twenge et al. (2001) found that simply leading participants to believe that they would end up alone in life showed reductions in self-regulatory capacities on experimental tasks—highlighting the need to believe in having lasting relationships. Work stemming from attachment theory—such as that by Mikulincer and Shaver (2007)—shows that enduring close relationships serve to buffer against stress and provide for well-being. Though there is no shortage of research on the importance of social relationships for well-being, few have explored specifically whether each relationship must meet all the criteria specified by Baumeister and Leary (1995) to fully satisfy one’s belongingness needs.

## Criteria for fully satisfying relationships and the problem with partial relationships

In closely examining Baumeister and Leary’s foundational review (1995), we have identified seven specific criteria for defining an interpersonal relationship as *fully satisfying* one’s belongingness needs. The criteria can be applied to evaluate each relationship a person has, and are as follows: one’s interactions with another should be (1) frequent, (2) affectively positive, and (3) relatively free of conflict; moreover, the person must share with the other individual (4) a common, stable sense of past history, (5) an expectation for future interactions, (6) affective concern for the other’s well-being, and (7) feel that their affective concern is mutually reciprocated. Although they argued that the first six criteria are essential, Baumeister and Leary (1995) expressed less certainty regarding the necessity of the seventh criterion. However, Clark et al. (1986) have shown that a framework of mutual concern produces qualitatively different relationships than those based on self-interested social exchange. Given this research, we included mutual affective concern as a seventh criterion to evaluate relationships as “fully satisfying.”

According to a strong version of Baumeister and Leary (1995) hypothesis, we argue that fully satisfying relationships with close others must meet *all seven* of the criteria listed above. We refer to such relationships as “whole” relationships whereas those that do not meet all seven criteria are termed “partial” relationships. Our terminology borrows from Baumeister and Leary (1995) who argued that *partial* relationships do not fully satisfy one’s belongingness needs, and that a minimum number of whole relationships are required. In fact, across two sections of their review on partial relationships they argued that belongingness needs are not fully satisfied when one has primarily (a) infrequent contact with those with whom they share meaningful connectedness or (b) frequent interactions with casual acquaintances (i.e., “without a bond of caring”).

Attempting to support the first of these two cases, Baumeister and Leary (1995) pointed to literature documenting the plight of prisoners who rarely see their family, noncustodial parents who are separated from their children, and couples engaged in long distance relationships. Relatively robust support for Baumeister and Leary (1995) first argument can be found in the unprecedented and severe social impacts wrought by the onset of COVID-19. Extended containment efforts, including school and university suspension of in-person learning, policies requiring “non-essential” employees to work remotely, and other isolation protocols drastically limited individuals’ opportunities to maintain meaningful connections with close others. Numerous studies documented sharp spikes in loneliness and social isolation during this time related to the widespread inability of individuals to connect with close others outside of the home (Farrell et al., 2023; Marmarosh et al., 2020).

Baumeister and Leary (1995) also highlighted the lack of fulfilling relationships among those who have frequent, but superficial relationship contacts. On this issue, there are numerous studies noting that individuals can feel lonely regardless of the quantity of their relationships (e.g., Mellor et al., 2008; Tsai and Reis, 2009; Green et al., 2001). Although nuanced, recent findings on the social impacts of online social media, which primarily facilitates myriad superficial interactions, also support Baumeister and Leary (1995) second argument. Having frequent superficial interactions via social media is shown to be a poor proxy for meaningful offline social connection (Marinucci et al., 2022), and in some cases, is associated with worsening experiences of social isolation and loneliness (Hunt et al., 2018; Primack et al., 2017).

## Meeting needs and diminishing returns

Baumeister and Leary (1995) also theorized that individuals require some minimum quantity of meaningful (i.e., whole) relationships. When the minimum quantity has not been met, the drive to form additional relationships or turn unsatisfying relationships into satisfying ones continues until the minimum is achieved. The strength of this drive is not expected to be the same for all individuals but instead should vary as a function of individual differences (Allen, 2025). Consistent with this claim, Kelly (2001) and Mellor et al. (2008) proposed that some people may have lower needs to belong that can be satisfied with a few contacts, whereas others with greater needs to belong require many contacts to sustain equivalent levels of satisfaction (i.e., it is not a “one size fits all” situation). Full examination of these ideas might include studies of individual differences in setting “minimum” thresholds or research on effortful activities consistent with a “drive” state. These issues are beyond the scope of the current paper that seeks merely to demonstrate that individuals require at least some small number of whole relationships to report high belongingness and well-being.

With respect to maximums, Baumeister and Leary (1995) surmised that once the minimum quantity of meaningful relationships is satisfied, not only does the motivation to establish additional relationships diminish, but the satisfaction of forming and the distress of terminating additional relationships are also diminished. Mellor et al. (2008) echoed this conclusion, stating that individuals who are well-enmeshed in social relationships should have less need to seek and form additional relationships than those who are socially deprived. An implication of this argument is that the relationship between numbers of whole relationships and perceived belongingness should follow a pattern of diminishing returns.

## Overview of present studies

To date, Baumeister and Leary (1995) influential paper on belongingness has been cited over 38,940 times. Nonetheless, an important facet of the belongingness hypothesis remains untested, namely, that specified criteria should be met for maintaining some minimum number of fully satisfying relationships. The following three studies explore this proposition. In the first of these studies, we report the development of a simple measurement scheme designed to assess the quantities of whole (i.e., fully satisfying) relationships reported by participants, and to differentiate these from quantities of partial (i.e., partially satisfying) relationships. This measurement scheme differs from standard instruments (e.g., social support measures) in that we ask individuals to evaluate “each” criterion for “each” of their close relationships, allowing the number of close relationships reported to vary across participants. We then use this measurement scheme to examine the hypothesis that greater numbers of whole relationships reported by individuals predict perceived belongingness and well-being (i.e., Hypothesis 1), whereas partial relationships do not have the same predictive utility (i.e., Hypothesis 2). Cutpoint criteria were determined based on a pilot study with 81 participants. We then tested the hypotheses using these criteria across three studies, adding two additional studies beyond the first to determine if the results replicate.

In each study, we examine a corollary of the hypothesis that individuals vary in the minimum number of whole relationships required to satisfy their belongingness needs. That is, if individuals require some small number of whole relationships to satisfy their belongingness needs, then there should be an upper limit such that additional relationships beyond the said limit provide no additional benefit. This is an issue of diminishing returns, and we will specifically examine the hypothesis that a curvilinear (logarithmic) relationship explains perceived belongingness and well-being as a function of the number of whole relationships reported (i.e., Hypothesis 3). As an exploratory analog to this hypothesis, we also examined whether there is a curvilinear relationship between the number of partial relationships reported and measures of belongingness and well-being (i.e., Research Question 1).

The first two studies also examine the hypothesis that the relation between whole relationships to subjective well-being is mediated through general perceived belongingness (i.e., Hypothesis 4). This mediational hypothesis is consistent with research by Mellor et al. (2008) who found evidence that loneliness mediates unmet belongingness needs in predicting life satisfaction.

Finally, Study 3 was designed to provide supplemental analyses to assess the contribution of each criterion to belongingness, as well as to replicate key findings from the first 2 studies. To conduct such an analysis required restructuring our questionnaires so that participants provided a single item assessment regarding how each relationship contributed to their belongingness needs. We did not use such an item in the first two studies to avoid priming participants with information linking the criteria to belongingness. Inserting the item in Study 3 allowed us to examine the contributions of each criterion to belongingness using multilevel modeling, where each relationship was nested within participants.

Before describing the details of these studies and the results, we note for those interested in these data for meta-analyses that the data set for Study 1was used by Pillow et al. (2015) to contrast Leary et al. (2013) Need to Belong Scale against Hagerty and Patusky (1995) Antecedents of the Sense of Belonging scale—both of which are measures of the need to belong. In addition, the first 2 data sets were used by Malone et al. (2012) in developing the General Belongingness Scale—describing its factor structure and correlates with other well-established measures. Neither of these papers provided a test of the belongingness hypothesis, examined correlations of whole vs. partial relationships with measures of achieved belonging, or assessed curvilinear effects.

## Study 1

The primary objective of the present study was to introduce a scheme for assessing quantities of whole vs. partial relationships, and to use this assessment to test the three hypotheses discussed above.

### Participants

The present sample (*N* = 872) was comprised of college students taking an introductory psychology course, and composed of 62% women, with an age range of 17 to 55 years (*M* = 19.30, *SD* = 3.14). Regarding ethnicity, 41% were Hispanic/Latino, 35% White, 10% Black/African American, 10% Asian/Pacific Islander, and 4% other. Data for this study and the following 2 studies were conducted with the approval of the appropriate institutional IRB. The data and syntax for Studies 1 through 3 can be found at https://.osf.io/hecqd.

### Procedure

Participants completed a set of questionnaires in a single session. Responses were collected online via SurveyMonkey software. At the inception of each study, the participants were asked to print out a table that was provided within the study or write on their own piece of paper a list of the: *“people that are very close to you.”* This method allowed the participants to keep track of the people very close to them without having to enter names of friends and relatives into the database. Before listing the names, the participants were instructed that there was no specific number of people that they should list. That is, the list could include zero, some, or many people; and that listing more or fewer people would not make the study any better or worse. The list was numbered from 1 to 14, allowing participants to report up to 14 relationships. The highest number of relationships reported in pretesting was 11 of the possible 14. Across the three studies reported in this paper, the largest quantity of participants who listed up to 14 relationships was only 2.4 percent, thereby indicating that the amount of space given to participants was acceptable. Participants subsequently completed questions for each relationship listed and a battery of other assessments as well. These assessments are described below.

### Measures

Measures were included to assess three overarching constructs: (1) quantities of whole vs. partial relationships; (2) sense of belonging; and (3) psychological well-being.

Belongingness Hypothesis Criteria Assessment. Participants were asked to answer a series of questions about each person that is *very close to them*. The first question asked: “*What is your relationship with this person?*” Twenty-four response options were included ranging from nuclear to extended family members, friends, neighbors, classmates, co-workers and others.

Subsequent items assessed the criteria for defining fully satisfying relationships. For example, one overarching criterion for defining a fully satisfying relationship is “that there is a need for frequent affectively pleasant interactions with a few people” (Baumeister and Leary, 1995, p. 497). Two facets of this criterion can be gleaned from the statement (i.e., frequency and positivity of interactions), and a third is implied and elaborated elsewhere in their review (i.e., free for a majority of the time from negativity; see p. 500). Moreover, the second overarching criterion that “these interactions must take place in the context of a temporally stable and enduring framework of affective concern for each other’s welfare” (p. 497) reflects what Baumeister and Leary (1995) envisioned in regard to relationship temporal stability, relationship continuing into the foreseeable future, and affective concern between individuals—with mutual affective concern as being ideal (see p. 500). Accordingly, the current research set out to define fully satisfying relationships using the following seven standards: (1) frequency of interactions, (2) positive interactions, (3) negative interactions, (4) temporal stability, (5) continuance into the foreseeable future, (6) affective concern, and (7) mutual affective concern. See Appendix A for the full instrument and cutpoints as discussed below.

*Cutpoints for defining fully satisfying relationships*. First, we generated items and rating scales for each of the standards. For a relationship to be classified as fully satisfying, or whole, the cutpoints across the entire set of standards must be met or exceeded. Relationships that did not meet or exceed the cutpoints across all seven standards were classified as partial relationships. This may seem a strict requirement, but the term *fully* indicates entirety, completeness, wholly, at least, sufficiency, and totality. Therefore, we argue that to define relationships as “whole” or “fully satisfying” when failing to meet all seven criteria would defy the basic meaning of the term as used by Baumeister and Leary (1995).

Cutpoints were based on both *a priori* and *post hoc* considerations to allow subjective judgments to converge with objective data (Violato et al., 2003). *A priori* cutpoints were established based on theoretical considerations of the belongingness hypothesis in combination with the characteristics of the present collegiate sample. Subsequently, analyses were conducted using a pilot sample of 81 persons to determine where to set the cutpoints to optimize the relationship between number of whole relationships reported with assessments of belonging and well-being. Specifically, we calculated the strength of the zero-order correlations between whole relationships and perceived belongingness to establish optimal cutpoints—where these were largely consistent with our *a priori* judgments. Summarizing, a triangulate approach for setting cutpoints was employed as follows: (1) the theory-based conceptualization of the belongingness hypothesis criteria, (2) qualifications given pretesting using a sample of university undergraduates, and (3) empirical tests of criterion-related validity (Fehrmann et al., 1991). *A priori* cutpoints are described as follows.

This initial set of standards was assessed using a frequency response format. First, Baumeister and Leary (1995) cited evidence that marital satisfaction declines among couples who see each other less than once a week (Gerstel and Gross, 1982). This provided a reasonable starting point for setting the frequency criterion to *once a week*. Furthermore, Baumeister and Leary (1995) argued that relationships should be free of negative affect or conflict most of the time. This led us to set the cutpoint for that standard at a frequency that occurs at least less than 50% of the time (i.e., *sometimes, seldom, almost never, or never*). On the other hand, though we expected close relationships to be relatively positive, it is not reasonable to conceive that positive interactions would always exist prompting us to set the cutpoint for positive interaction at *often* (and including *almost always* and *always*). In addition, the cutpoint for continuance of relationship into the foreseeable future was set at *10 to 20 years* so that we might ensure that participants (i.e., undergraduates) view reported relationships as continuing beyond their tenure in college.

The remaining three standards were assessed based on level of agreement. For each of these standards, we took a moderately conservative approach and set the initial criteria at the next to the highest level possible to define fully satisfying relationships. Specifically, the temporal stability, affective concern, and mutual affective concern standards were set at one level below *strongly agree* (i.e., *agree*).

#### Assessments of perceived belongingness and lack of belongingness

*General Belongingness.* The 12-item General Belongingness Scale (GBS) assesses a general belongingness across friends and family, social others, and beyond interpersonal relationships (Malone et al., 2012). The GBS was rated on a 7-point scale ranging from 1 (*strongly disagree*) to 7 (*strongly agree*). Negative-worded items were reverse-scored. Higher scores indicate greater general belongingness.

*Sense of Belonging.* The 18-item Sense of Belonging Instrument-Psychological Experiences (SOBI-P) assesses a sense of belonging (Hagerty and Patusky, 1995). The SOBI-P was rated on a 4-point scale ranging from 1 (*strongly disagree*) to 4 (*strongly agree*). Negative-worded items were reverse-scored. Higher scores indicate a greater sense of belonging.

*Loneliness.* The 8-item short-form of the UCLA Loneliness Scale (ULS-8) assesses how much a person feels cut off or separated from others. These feelings can result from either a deficit in the quantity or quality of social contacts (Hayes and DiMatteo, 1987). The ULS-8 was rated on a 4-point scale ranging from 1 (*often*) to 4 (*never*). Loneliness-directed items were reversed-scored. Higher scores indicate greater levels of loneliness.

#### Assessments of positive and negative well-being

*Life Satisfaction.* The 5-item Satisfaction with Life Scale (SWLS) assesses a person’s global satisfaction with life (Diener et al., 1985). The SWLS was rated on a 7-point scale ranging from 1 (*strongly disagree*) to 7 (*strongly agree*). Higher scores indicate greater satisfaction with life.

*Happiness.* The 4-item Subjective Happiness Scale (SHS) assesses a person’s global subjective happiness (Lyubomirsky and Lepper, 1999). The SHS was rated on a 7- point scale ranging from 1 (*less happy*) to 7 (*more happy*) and 1 (*not at all*) to 7 (*a great deal*). Higher scores indicate greater levels of subjective happiness.

*Depression.* The 20-item Center for Epidemiologic Studies Depression Scale (CES-D) was primarily designed to assess depressive symptomatology in the general public (Radloff, 1977). To emphasize current state, the directions read, “*How often this past week did you*…” The CES-D is scored using a 0–3 scoring system. Items assessing positive affect were reverse-scored. Higher scores indicate greater frequency, which equate to more depressive symptoms.

Reliability of Measures of Belonging and Well-being. Coefficient *alphas* for the six measures described above ranged from 0.85 to 0.96 across Studies 1 through 3.

### Results and discussion

#### Descriptive statistics

College students reported a median of four very close relationships. Across participants, a total of 3,693 relationships were reported. Of those relationships reported, friends accounted for the highest total (34%) followed by mothers (17%), siblings (15%), fathers (11%), girl/boyfriends (9%), extended family members (9%) and others (5%).

#### Preliminary analyses

All analyses reported in this paper were performed using SPSS Statistics (Version 30, IBM Corp., 2024) and SPSS Amos (Version 29, IBM Corp., 2022).

Whole and Partial Relationship Descriptives. Using the final cutpoints for the seven standards described in the method section, we calculated numbers of whole and partial relationships for each participant in the study. Each relationship listed by participants that met or exceeded all seven of the criteria was counted as a whole relationship (i.e., fully satisfying relationships); whereas each relationship listed by participants that failed to meet one or more criteria was counted as a partial relationship (i.e., partially satisfying relationships).

An examination of the descriptive statistics revealed that slightly more than half of the relationships were defined as whole relationships and slightly less than half as partials (*M*_whole_ = 2.42, *SD* = 2.05; and *M*_partial_ = 1.79, *SD* = 1.99). As indicated in Figure 1, it was found that approximately 14.8% of students reported having zero whole relationships and approximately 78.1% reported having from 1 to 5. Similarly, it was found that approximately 30.3% reported zero partial relationships and 63.7% reported from 1 to 5. Zero-order correlations for variables used in the models for Studies 1, 2, and 3 are shown in Supplementary Appendix C, Tables 1–3.

**Figure 1:**
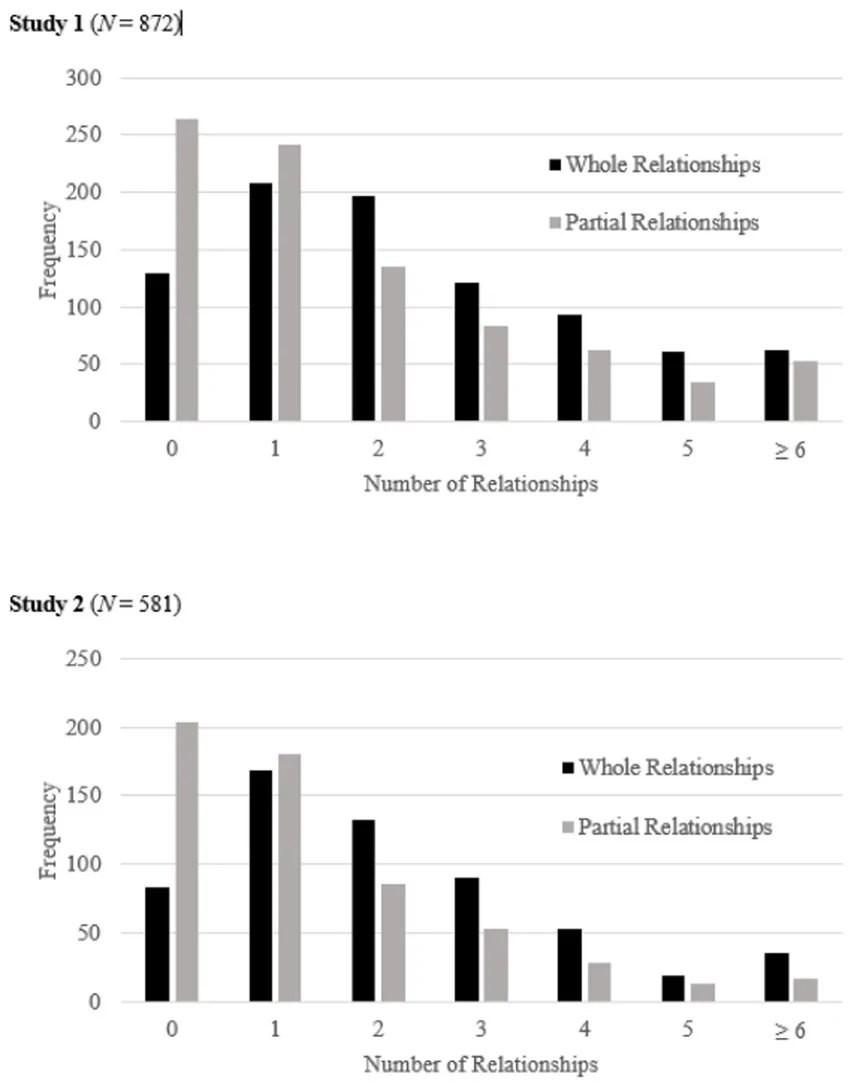
Frequency counts by relationship type across Studies 1 and 2.

Overview of the Analytical Model Used for Hypothesis Testing. We used structural equation modeling as a framework to test each of our three major hypotheses. The initial analytical model is displayed in Figure 2. As shown, belonging and well-being served as the criterion variables in the study and specified as latent constructs, where each construct was represented by multiple indicators. Quantity of whole and partial predictors were added next to test our primary hypotheses. Although the model was initially estimated with all variables included as shown in Figure 2, specific pathways were evaluated sequentially and separately as subsequently explained.

**Figure 2:**
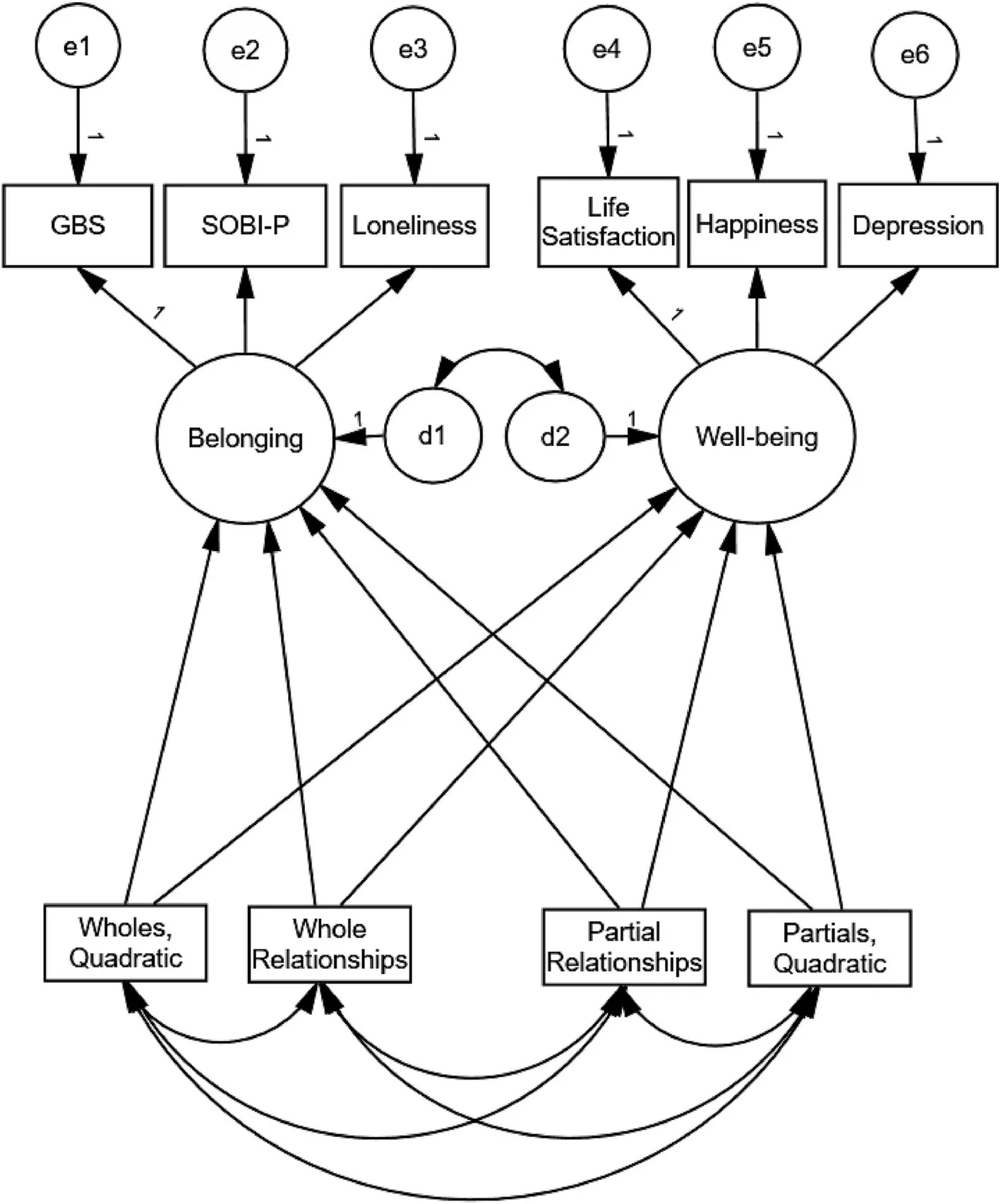
(*N* = 872). Fully saturated model. GBS, General Belongingness Scale; SOBI-P, Sense of Belonging Instrument – Psychological Experiences. Relationship types predict belonging and well-being. Quadratic terms indicate diminishing returns.

Overall Model Fit. The model—as shown in Figure 2—was found to fit the data very well, *χ*^2^ (24) = 77.48 (*p* < 0.001), Comparative Fit Index (CFI) = 0.990, root mean square of the error approximation (RMSEA) = 0.051, 90% Confidence Intervals (CI) = 0.038–0.063. The estimates from the model shown in Figure 2 were then used to test our first 3 hypotheses. The correlation matrix and descriptives for all three studies reported in this paper can be found in the Supplementary materials.

Test of Hypothesis 1: Do whole relationships predict belongingness and well-being?

To test this hypothesis, we used estimates from the model based on Figure 2 and examined the effects of the number of whole relationships on the latent variables of belongingness and well-being. As predicted, the number of whole relationships reported by participants was positively associated with perceived belongingness and well-being, both βs=.35, ps<.001 (see Table 1 for unstandardized estimates and details). As indicated by the standardized path coefficients, these relationships are moderate in size.

**Table 1:** Coefficients and significance tests predicting belongingness and well-being as a function of quantities of whole and partial relationships across Studies 1, 2, and 3.

	Belonging				Well-being			
	*β*	B	SE	CR	*β*	B	SE	CR
Hypothesis 1: Numbers of whole relationships positively predict outcomes.								
Whole Relationships^1^	0.350	0.189	0.023	8.11^***^	0.353	0.188	0.025	7.56^***^
Whole Relationships^2^	0.311	0.139	0.025	3.91^***^	0.231	0.123	0.031	3.91^***^
Whole Relationships^3^	0.433	0.174	0.033	5.21^***^	0.424	0.178	0.038	4.66^***^
Hypothesis 2: Numbers of partial relationships do not predict outcomes like wholes.^4^								
Partial Relationships^1^	−0.124	−0.069	0.027	−2.57^*^	−0.259	−0.142	0.028	−5.02^***^
Partial Relationships^2^	−0.225	−0.108	0.029	−3.66^**^	−0.268	−0.152	0.037	−4.16^***^
Partial Relationships^3^	−0.190	−0.083	0.038	−2.19^*^	−0.163	−0.074	0.042	−1.75^†^
Hypothesis 3: There is a pattern of diminishing returns for whole relationships.								
Wholes, Quadratic^1^	−0.139	−0.017	0.005	−3.21^**^	−0.125	−0.015	0.005	−2.72^**^
Wholes, Quadratic^2^	−0.076	−0.009	0.006	−1.35	−0.052	−0.007	0.008	−0.90
Wholes, Quadratic^3^	−0.169	−0.015	0.007	−2.11^*^	−0.130	−0.012	0.008	−1.51
Exploratory: Is there a quadratic effect for partial relationships on belonging and well-being?								
Partials, Quadratic^1^	0.052	0.007	0.006	1.09	0.160	0.020	0.006	3.13^**^
Partials, Quadratic^2^	0.072	0.007	0.006	1.18	0.146	0.016	0.007	2.29^*^
Partials, Quadratic^3^	0.166	0.002	0.008	1.89^†^	0.119	0.014	0.009	1.45

Study 1 (*N* = 872); Study 2 (*N* = 581); Study 3 (*N* = 284); (^1^Study 1; ^2^Study 2; ^†^*p* < 0.10; ^*^p < 0.05; ^**^p < 0.01; ^***^p < 0.001. ^4^Hypothesis 2 is formally tested by a chi-square difference test as presented in text, and those difference tests are significant for each study.

Test of Hypothesis 2: Do whole relationships predict differently than partial relationships? Hypothesis 2 was a corollary of Hypothesis 1 and was again evaluated based on estimates of the model shown in Figure 2. To test the hypothesis that the effects of whole relationships and partial relationships are not equivalent, we imposed equality constraints on the paths from whole vs. partial relationships to belongingness and well-being. Using chi-square difference testing, the pathways from whole and partial relationships to belongingness ( βs=.35vs.−.12) were found to differ significantly, ∆*χ*^2^ (1) = 55.46, *p* < 0.001. Not only was the association between partial relationships and belongingness weaker compared to that for whole relationships, but the association was found to be negative, *β* = −.12, *p* = .05. A second equality constraint was added to the model requiring that the paths from whole and partial relationships to well-being be equal. Again, the paths were found to be unequal in the expected direction, ∆*χ*^2^ (1) = 82.07, *p* < 0.001. A significant, inverse association between number of partial relationships and well-being was found, indicating the greater the number of partial relationships reported the less one’s well-being, *β* = −0.26, *p* < 0.001. These results provide strong support for our hypothesis that whole relationships function differently than do partial relationships in predicting positive outcomes for individuals. Although not expressly predicted, we found not only that partial relationships fail to contribute positively to perceived belongingness and well-being, but evidence indicating they detract from them.

Test of Hypothesis 3: Is there evidence that whole relationships contribute via a pattern of diminishing returns? Hypothesis 3 was that, on average, individuals need at least one or more whole relationship to satisfy their belongingness needs and sense of well-being. The contribution of the additional whole relationships beyond this minimum is hypothesized to yield diminishing returns evident in the form a logarithmic relationship. Thus, we predicted that a quadratic relationship would modify the expected linear relationship stated in Hypothesis 1. To test this hypothesis, we constructed a quadratic term representing the number of whole relationships by squaring the centered measure of whole relationships.

As shown in Table 1, statistically significant quadratic effects for belongingness and well-being were obtained modifying the linear effects, *β* = −0.14 (*p* = 0.001), *β* = −0.13 (*p* < 0.01), respectively. Focusing on belonginess as the DV, the negative coefficient on the quadratic term describes a concave downward curve as illustrated in Figure 3. This figure shows the unconditioned quadratic relationships after using the factor score regression weights to reproduce the factor scores representing the latent variables. To maximize interpretability, the factor scores representing belongingness and well-being have been rescaled using the means and standard deviations based on the 7-point scaling of the General Belongingness Scale and the Happiness Scale, respectively.

**Figure 3:**
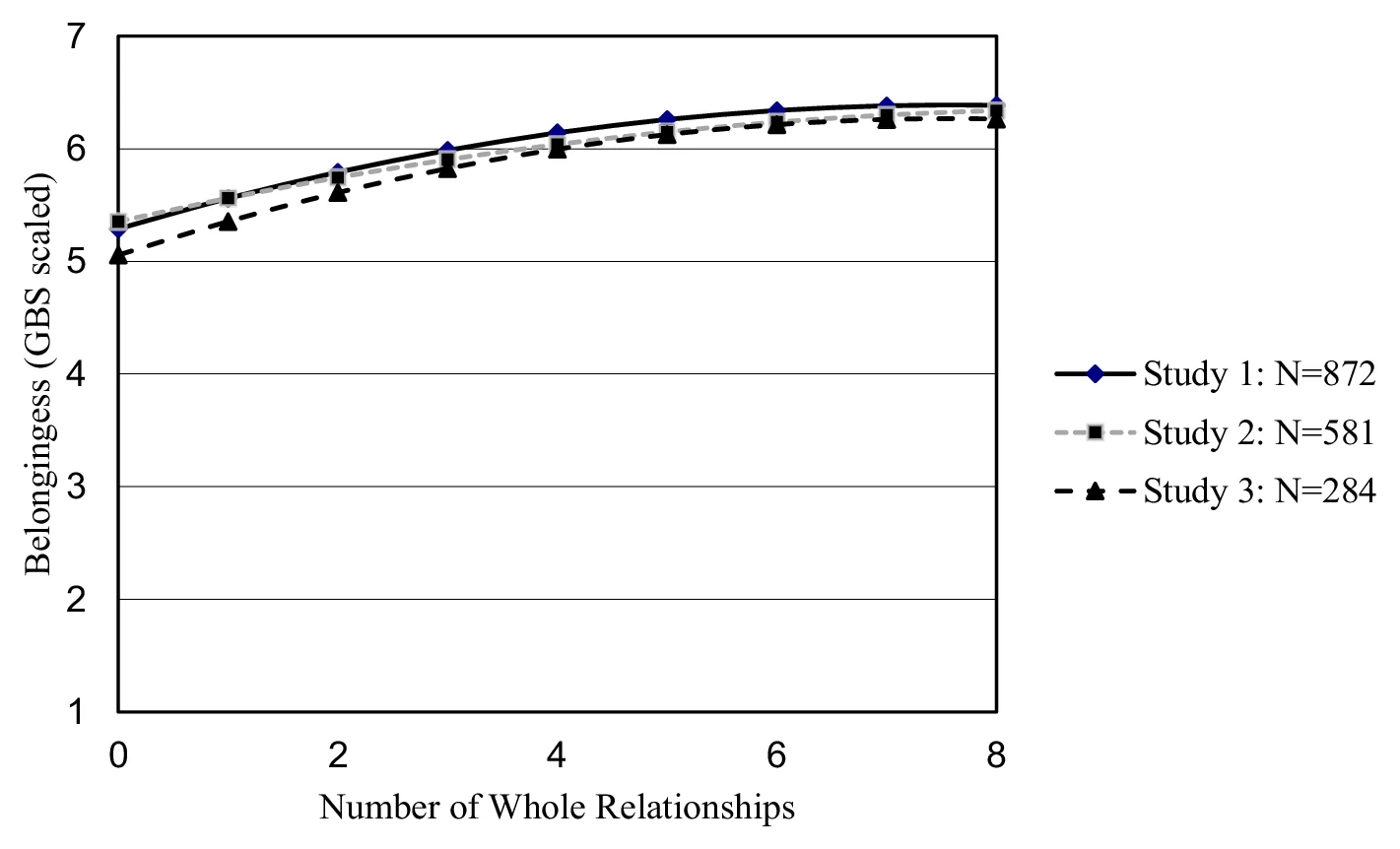
Study 1 (*N* = 872); Study 2 (*N* = 581); Study 3 (*N* = 284). GBS, General Belongingness Scale. Whole relationships show diminishing returns in belongingness—though the quadratic effect is not significant improvement over the linear effect in Study 2. Whole relationships show marked *increases* in belongingness for persons with 1 to 3 whole relationships relative to 0, but then the relationship begins to flatten out at numbers greater than or equal to four.

As illustrated in the left-hand side of Figure 3, the largest increase in perceived belongingness is evident when comparing those with zero (or no) whole relationships against those with one or two such relationships. This is bolstered by simple mean comparisons (via an ANOVA with Tukey post-hoc corrections) of those reporting 0, 1, 2, 3, or 4 or more whole relationships, where the means equal $5.21_{a}$ , $5.57_{b}$ , $5.84_{\mathrm{bc}}$ , $6.02_{\mathrm{cd}}$ , and $6.20_{d}$ , respectively. The largest difference is between those reporting 0 whole relationships and those reporting 1, and then the differences become progressively smaller. All pairwise comparisons sharing the same subscript are not significantly different from one another.

Although not hypothesized *a priori*, parallel quadratic effects (constructed by squaring the centered number of partial relationships) were examined with respect to quantities of partial relationships reported by participants (i.e., Research Question 1). The quadratic effect relating partials to belongingness was not statistically significant, *p* = 0.274. However, the quadratic effect relating partials to well-being was significant, *β* = 0.16, *p* = 0.002. Here again, there is a pattern of diminishing returns, but one that operates in the inverse direction. Participants reporting one or two partial relationships tend to be significantly lower on measures of well-being than those reporting zero partial relationships. On the other hand, decrements in well-being decline with increasing numbers of partial relationships (see Figure 4).

**Figure 4:**
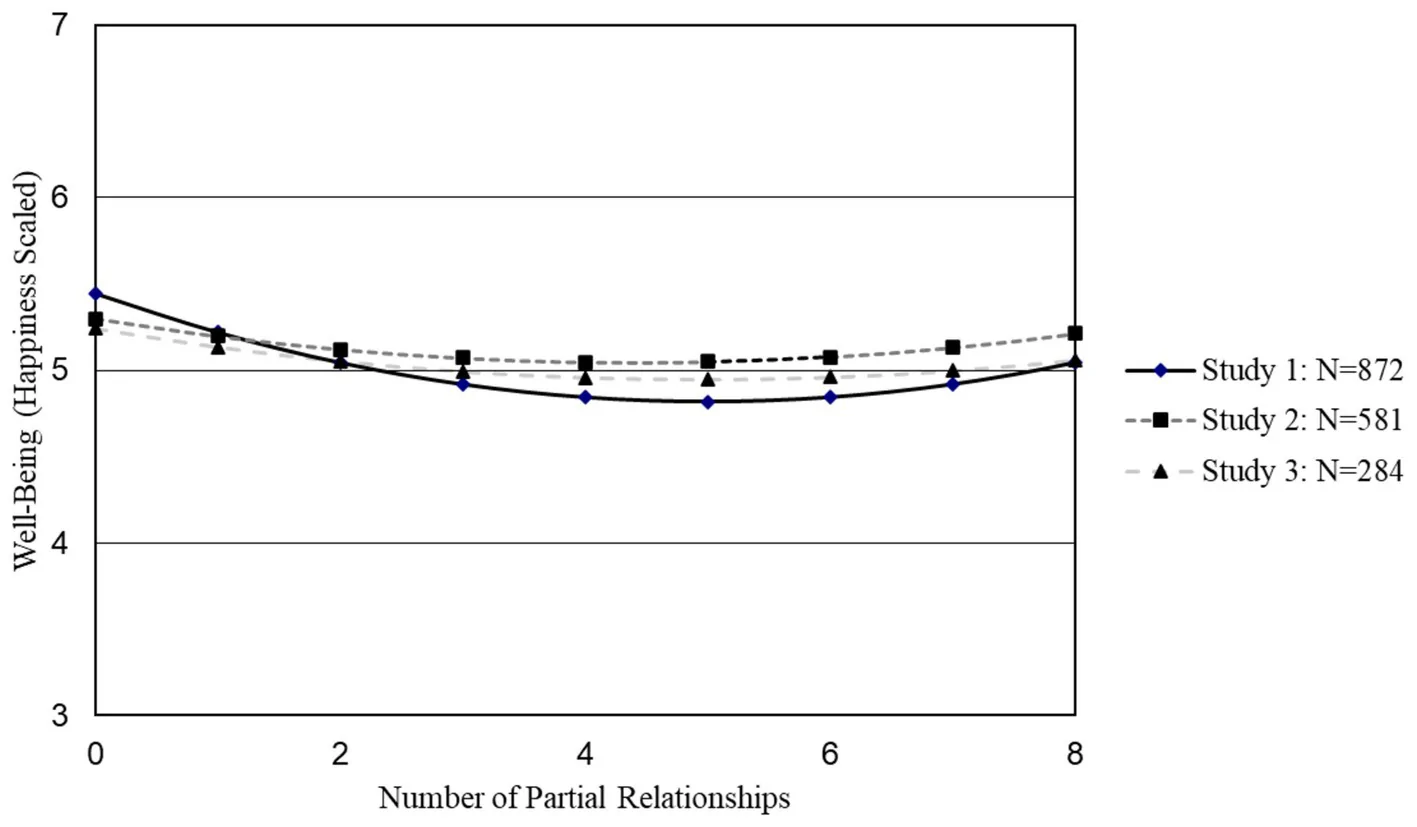
Study 1 (*N* = 872); Study 2 (*N* = 581); Study 3 (*N* = 284). In Studies 1 and 2, partial relationships show diminishing returns with well-being. Partial relationships show marked *decreases* in well-being across numbers zero through three but then begin to flatten out at numbers greater than or equal to four. Study 3 displays the same pattern, but the slope is reduced when controlling for the effect of whole relationships and becomes non-significant (i.e., it is statistically significant when entered in a reduced model).

Hypothesis 4 and Summary Model. After testing each of the three major hypotheses, we summarized the results by eliminating pathways involved in hypothesis testing that were not statistically significant at p<.05. To construct an optimally parsimonious model and test for mediational effects, we included a directional path from belongingness to well-being based on prevailing theory that regards belongingness as a need that must be met if humans are to be happy and satisfied (Baumeister and Leary, 1995; Fiske and Yamamoto, 2005). This re-specification resulted in dropping the nonsignificant pathways representing the linear and quadratic effects of whole relationships on well-being, consistent with the idea that these effects are mediated through belongingness. Model fit was very good, *χ*^2^ (27) = 83.26 (*p* < .001), CFI = .989, RMSEA = .049, 90% CI[.037, .061], *pclose* = .538. The indirect tests were found to be statistically significant, $\beta_{\text{indirect}}=.310\;(\mathrm{SE}=.037),95\%\mathrm{CI}\;[.235,.383]$ , for the linear relationship, and $\beta_{\text{indirect}}=-.124\;(\mathrm{SE}=.032),95\%\mathrm{CI}\;[-.187,-.061]$ for the quadratic relationship (see Figure 5). In addition, a significant indirect effect was obtained linking partial relationships to well-being through belongingness, $\beta_{\text{indirect}}=-.074\;(\mathrm{SE}=.028),95\%\mathrm{CI}\;[-.133,-.019]$ . Thus, partial relationships had a negative effect on well-being despite lacking a significant direct effect.

**Figure 5:**
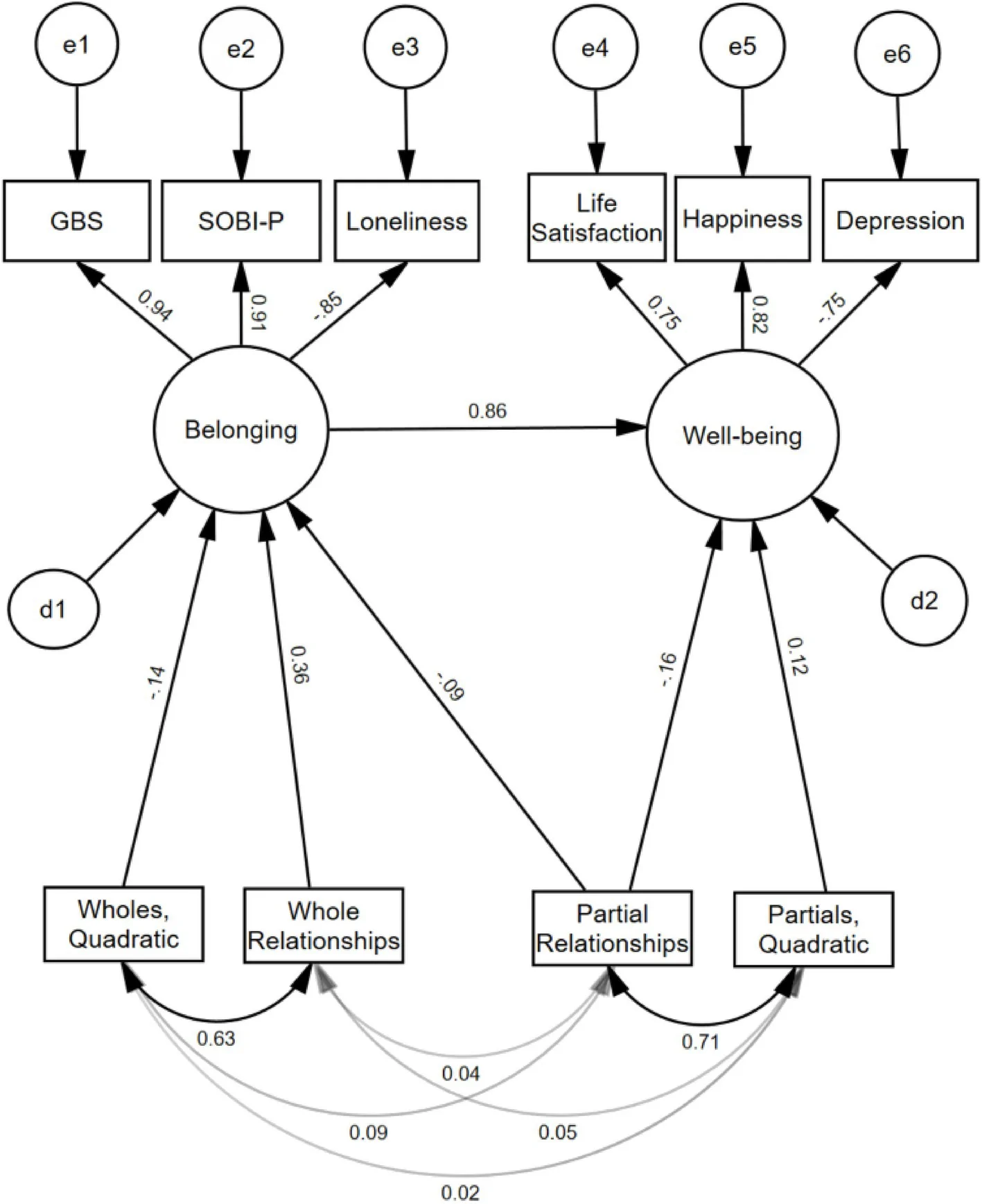
(*N* = 872). GBS, General Belongingness Scale; SOBI-P, Sense of Belonging Instrument – Psychological Experiences. Quadratic terms indicate diminishing returns. Coefficients are standardized. Light-faced curves showing correlations among the exogenous variables are not statistically significant. All other coefficients shown are statistically significant at less than 0.05. All coefficients are standardized.

## Study 2

The primary objective of the present study was to replicate the findings of Study 1 on a new sample. The cutpoints used in Study 2 were the same as those used in Study 1.

### Method

#### Participants

The present sample of college students (*N* = 581) was comprised of 54% females, with an age range of 17 to 50 years (*M* = 19.67, *SD* = 2.93). Regarding ethnicity, 42% were Hispanic, 39% White/Caucasian, 9% Black/African American, 7% Asian/Pacific Islander and 3% other.

#### Procedure

The procedure of the present study replicated that of Study 1. For a detailed description, see the procedure section of Study 1.

#### Measures

The following instruments were used in the previous study and again in the present study. Information regarding the reliability and validity of the following instruments can be located in the Measures section of Study 1: General Belongingness Scale (GBS); Sense of Belonging Instrument-Psychological Experiences (SOBI-P); short-form of the UCLA Loneliness Scale (ULS-8); Satisfaction with Life Scale (SWLS); Subjective Happiness Scale (SHS); and the Center for Epidemiologic Studies Depression Scale (CES-D). There were 2 minor wording and order differences in the GBS detailed in Appendix B.

### Results and discussion

#### Whole and partial relationship descriptives

Descriptive statistics across Studies 1 and 2 were remarkably similar. In the present study, participants reported a total of 2,108 relationships with friends again accounting for the highest total (30%) followed by mothers (19%), siblings (15%), fathers (11%), girl/boyfriends (11%), extended family (7%) and others (7%). Whole relationship (*M*_whole_ = 2.18, *SD* = 1.84) and partial relationship (*M*_partial_ = 1.42, *SD* = 1.72) means for the two studies were also comparable, with only small differences ( $M_{\text{Whole Diff}}=0.24$ and $M_{\text{Partial Diff}}=0.37)$ . In addition, the overall distributions of numbers of whole and partial relationships reported across Studies 1 and 2 were generally similar. See Figure 1 for a frequency distribution of whole and partial relationships.

#### Replication of the hypothesis tests conducted in study 1

Model fits for the previous study were replicated in this present study. With the structural portion of the model fully saturated to facilitate hypothesis testing via methods used in Study 1, the overall model provided an acceptable fit to the data, *χ*^2^ (24) = 89.49, CFI = 0.976, RMSEA = 0.069, 90% CI = 0.054–0.084.

Each of the three hypothesis tests examined in Study 1 were replicated and supported here in Study 2. First, confirming Hypothesis 1, whole relationships showed moderately strong positive associations with belongingness and well-being, βs=.31 and .23 , respectively, ps<.001 (see Table 1 for details).

Second, confirming Hypothesis 2, higher numbers of whole relationships were found to differ from partial relationships in how strongly they predicted higher levels of belongingness and well-being. This was verified by chi-square difference tests conducted by imposing equality constraints for belongingness, $\Delta \chi^{2}(1)=44.75$ , and well-being, ∆χ^2^ (1) = 36.68, *p*s < 0.001. Again, higher numbers of partial relationships were inversely related to belongingness and well-being, and in the present study these negative relationships were clearly statistically significant (see Table 1).

Third, evidence for Hypothesis 3 stating that numbers of fully satisfying relationships contribute to perceived belongingness via a pattern of diminishing returns was not supported in Study 2. The quadratic term for whole relationships to belongingness trended in the expected direction but was not statistically significant β=−.08,p<.18 . Similarly, the quadratic term predicting well-being was not statistically significant, β=−.05,p=.37 (see Table 1). This is a failure to replicate Study 1. However, what this analysis indicates is that the quadratic effect is not a significant improvement in fit over the linear—rather than a disconfirmation of the diminishing returns pattern. Figure 6 shows curves fit to the data pattern linking whole relationships to belonging (on top) contrasted against the number of total relationships predicting belongingness (on bottom). The most robust part of the pattern is the increase in the belongingness for individuals who have 1, 2, and then 3 whole relationships compared to none. In fact, the linear slope for just this part of the data is b=.19,p<.001 , where the linear slope for those with 4 or more whole relationships is b=.057,p=.23 . In summary, the data are consistent with both a linear effect and a pattern of diminishing returns—though importantly, the quadratic effect does not yield a statistically significant improvement over the linear function, and again, fails to confirm Hypothesis 3.

**Figure 6:**
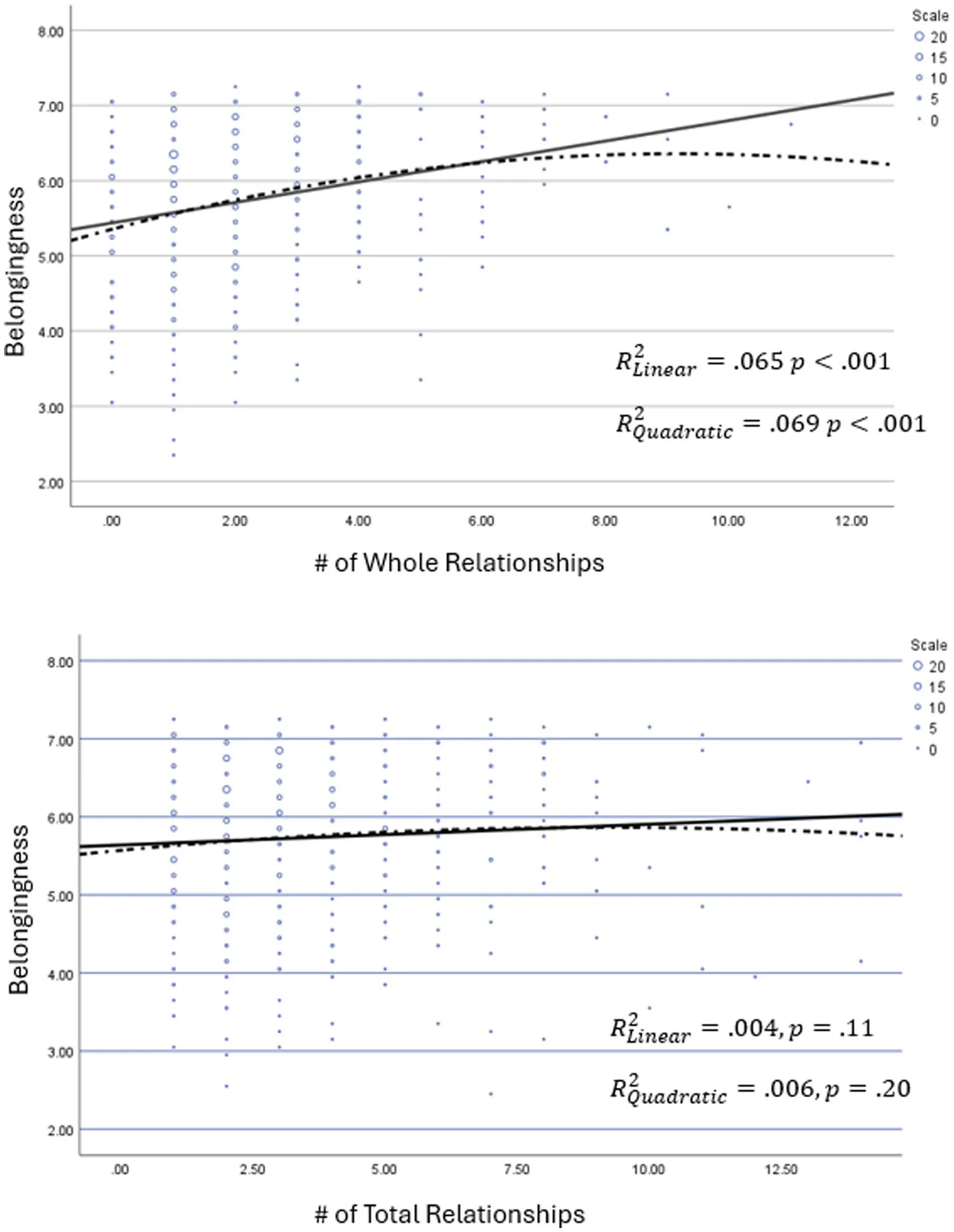
(*N* = 581). Whole relationships are related to perceived belongingness whereas the total number of close relationships are not. The top figure shows that the linear effect and quadratic effect are virtually indistinguishable in Study 2.

Patterns of diminishing returns were replicated for partial relationships in predicting well-being (see Figure 4). Here, the first few partial relationships contribute substantially to the quadratic term supporting this conclusion and is statistically significant, β=.146,p=.022 . Consistent with Study 1, there was no significant quadratic for partial relationships predicting belongingness (see Table 1).

#### Hypothesis 4 and summary model

After testing each specific hypothesis, we summarized the model by eliminating nonsignificant pathways (i.e., paths where p>.05 excepting the key path from partial relationships to well-being to clarify key hypothesized relationships and differences across studies). The model fit was acceptable, χ^2^ (27) = 91.58, CFI = 0.976, RMSEA = 0.064, 90% CI = 0.050–0.079. Consistent with Study 1, we included a directional path from belongingness to well-being and again found that the linear effect of belongingness was significantly related and quadratic terms for whole relationships were mediated by belongingness in predicting well-being. The indirect test for the linear relation was statistically significant, $\beta_{\text{indirect}}=.17\;(\mathrm{SE}=.047),95\%\mathrm{CI}\;[.17,.35]$ , but was not statistically significant for the quadratic effect, $\beta_{\text{indirect}}=-.07\;(\mathrm{SE}=.039),95\%\mathrm{CI}\;[-.15,.01]$ (see Figure 7). The indirect effect linking partial relationships to well-being was significant, $\beta_{\text{indirect}}=-.146\;(\mathrm{SE}=.040),\;95\%\mathrm{CI}\;[-.23,-.07]$ for the quadratic relationship (see Figure 7). Overall, the data supports Hypothesis 4—finding most importantly that the numbers of whole relationships on well-being can be explained through belongingness.

**Figure 7:**
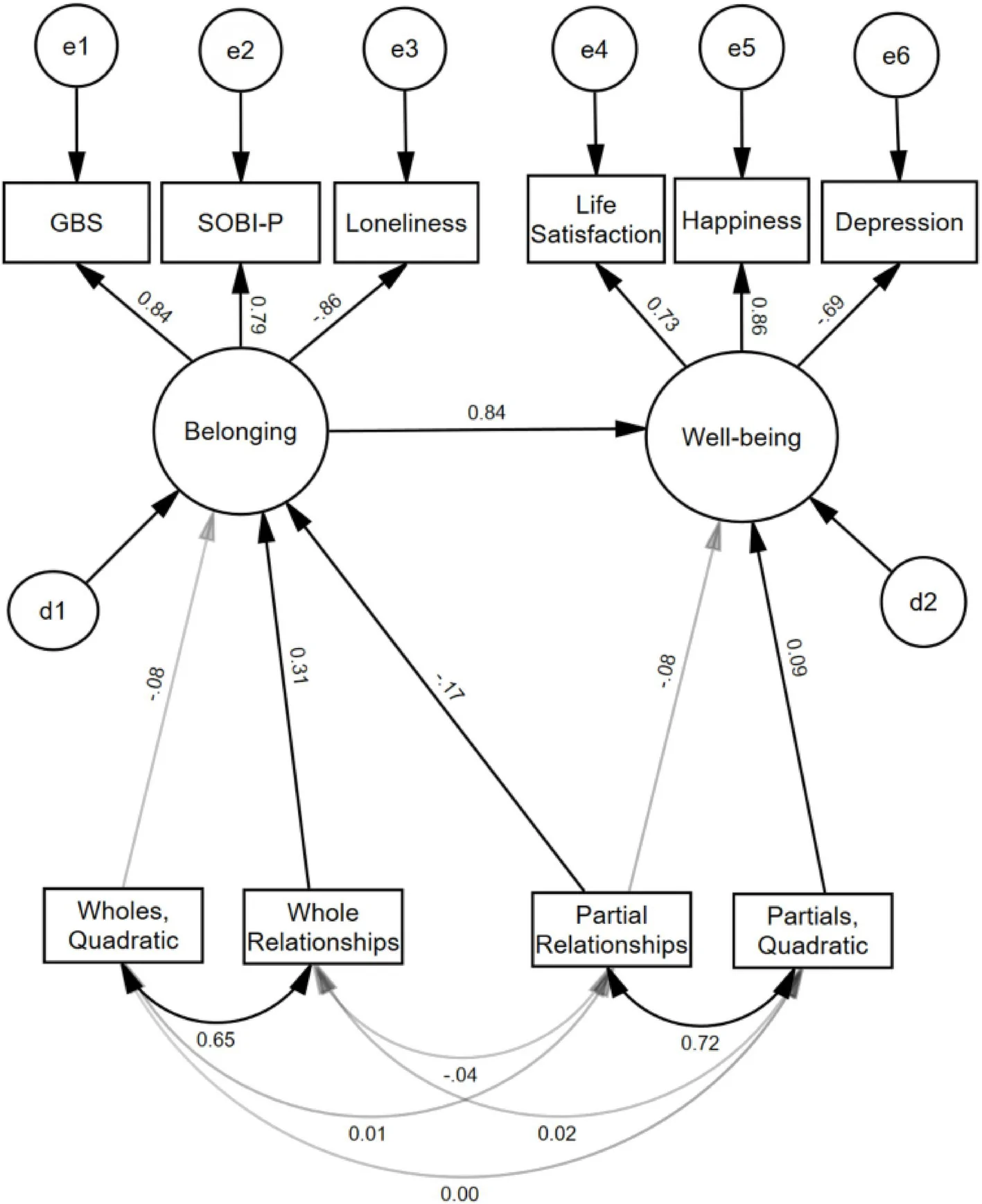
(*N* = 581). GBS, General Belongingness Scale; SOBI-P, Sense of Belonging Instrument – Psychological Experiences. Quadratic terms indicate diminishing returns. Coefficients are standardized. Light-faced curves showing correlations among the exogenous variables and light-faced structural paths are not statistically significant. All other coefficients shown are statistically significant at less than 0.05.

### Discussion

The result of Study 2 confirmed the results of Study 1 with respect to three of our four hypotheses. Our first two studies established theoretically derived criteria for evaluating each close relationship participants reported. This method enabled a unique examination of the data linking close relationships to belongingness and well-being: namely, it allowed for an assessment of what minimum number of relationships are needed to meet belongingness needs, with both studies showing that the first 3 whole relationships provide for substantial increases in perceived belongingness. We subsequently conducted a third study to again see if the patterns from Study 1 replicate and to further examine how these criteria might be both interrelated and operate at the relationship level.

## Study 3

One limitation of the first two studies is that belongingness and well-being were assessed at the level of the individual whereas the criteria separating wholes from partials were assessed at the relationship level of analysis. That is, the close relationships reported by participants were nested within individuals—where each participant reported on 0 to 14 relationships (though only 2 participants across the first two studies reported 0 close relationships). This design structure served two purposes. First, the strategy was consistent with our plan to examine how belongingness varies as a function of the numbers of whole relationships reported—using information at the relationship level to construct tallies at the person level. Second, and most importantly, we did not assess belongingness at the relationship level to avoid making our hypothesized links to belongingness less salient (i.e., reduce the possibility of an expectancy effect that could bias the results). These design features, however, preclude examining how the seven criteria relate to perceptions of belongingness at the relationship level.

In contrast, this current study included a single rating of belongingness for each relationship reported by participants. This methodological alteration permits the use of a multilevel framework to examine how the belongingness criteria contribute additively and via interactions in explaining perceived relationship belongingness. Notably, Baumeister and Leary (1995) claimed that a fully satisfying relationship requires *both* high frequency of interactions and a sense of connectedness (per the remaining 6 criteria such as stability and affective concern)—implying a statistical interaction. To conduct an analysis that examines whether frequency of human interactions statistically interacts with other criteria requires first dealing with expected multicollinearity as some of the criteria are likely to be highly correlated (e.g., concern and mutual concern). Thus, we approached Study 3 by first conducting a factor analysis to examine how the criteria are interrelated with respect to underlying constructs. This should then facilitate examining interactions of frequency with factors representing the remaining six criteria. The second analysis step planned involved regressing perceived relational belongingness onto the underlying constructs representing the seven criteria and forming the interactions of the constructs. Prior to these analyses, however, we examine whether the data from Study 3 replicates the findings of the first two studies.

### Method

#### Participants

The present sample of college students (*N* = 284) was comprised of 61% females, with an age range of 17 to 45 years (*M* = 20.3, *SD* = 3.8). In regard to ethnicity, 47% were Hispanic, 34% White/Caucasian, 9% Black/African American, 7% Asian/Pacific Islander, and 3% other.

#### Measures

All methods and measures were kept the same as in the previous two studies excepting one alteration. When assessing the 7 standards for assessing whole relationships, we added one item to assess perceptions of belonging for each relationship reported. Specifically, participants were asked the extent to which they agreed with the statement, “This person provides me with a sense of belonging.” Participants responded on a 9-point Likert scale that ranged from, “Very Strongly Agree” to “Very Strongly Disagree.” This method allowed us to assess the association between each relationship and to what extent that person provided a sense of belonging.

### Results and discussion

#### Replication of studies 1 and 2

Prior to reporting the multilevel findings, we first examined whether the results supported the hypotheses tested in Studies 1 and 2. Consistent with the first two studies, we estimated the model shown in Figure 2 using the new data set. Again, the model was found to have a excellent fit, *χ*^2^ (24) = 37.17 (*p* = .04), CFI = .991, *RMSEA* = .044, 90% CI[.009, .07], *pclose* = .61. As shown in Table 1, Hypothesis 1 was confirmed, with numbers of whole relationships predicting greater belongingness and well-being. Hypothesis 2 examined whether the coefficients linking partial relationships to belongingness and well-being ( −.19,−.16 ) were equal to those linking whole relationships to belongingness and well-being ( .43,.42 ). Chi-square difference tests examined Hypothesis 2 by imposing equality constraints thereby confirming that the structural coefficients were different with respect to both belongingness, $\Delta \chi^{2}(1)=32.07$ , and well-being, $\Delta \chi^{2}(1)=24.92,\mathrm{ps}<.001$ . Again, the coefficient linking partial relationships to belongingness was not only different from those of whole relationships but was significantly negative (see Table 1). The negative relation of partial relationships to well-being was not significant, but trended toward it, p=.08 . As shown in Table 1 and displayed in Figure 3, support was obtained for Hypothesis 3 that predicted a pattern of diminishing returns in belongingness as numbers of whole relationships increase. This replicates Study 1. On the other hand, the effect for well-being was not significant. As for the exploratory question about the curvilinear effect of partial relationships, the effect for well-being was not replicated (see Table 1 and Figure 4).

#### Preliminary analyses

We initially conducted a factor analysis of the seven belongingness criteria to reduce the number of predictors to be used in exploring main effects and interaction effects in the multilevel analysis. The exploratory factor analysis was conducted using Maximum Likelihood Estimation with a Promax rotation in SPSS. An initial 2-factor solution was found accounting for 44.4% of the variance in the measures. The loadings on these factors were healthy with one exception: frequency of interactions did not load above 0.3 on either factor. In comparison, a 3-factor solution was found to account for 58.31% of the variance and was found to fit the data well via a goodness of fit test that yielded a nonsignificant Chi-square (3) of 7.36, *p* = 0.061. In this solution, ‘Frequency of Interactions’ defined one factor by itself and was labeled as such. The second factor was labeled ‘Positive/Stable Interactions’ with standardized factor loadings as follows: positive interactions = 0.91, negative interactions = −0.70, and stability = 0.51. The third factor was labeled, ‘Enduring Concern’ with standardized loadings as follows: affective concern = 0.73, mutual affective concern = 0.76, and the expectation that the relationship will continue into the foreseeable future = 0.53. The resulting factor scores were saved into the data set using the factor analysis module, all with means equal to 0. These scores, already centered, were then multiplied together to form variables representing the three two-way interactions and the one three-way interaction term.

A random intercepts model was first estimated to determine the amount of variance in perceived belongingness that was explained at the relationship level vs. the person level. There was significance variance in the intercepts representing the means at the person level, estimate = 0.465, *z* = 7.05, *p* < 0.0001. The ICC was 0.31, indicating that 31% of the variance in ratings of relational belongingness could be attributed to individual differences between participants in the study. The remaining 69% of the variance was due to differences in relationships nested within participants (Table 2).

**Table 2:** Multilevel within-person coefficients and significance tests predicting the perceived belonging per relationship as a function of subsets of whole relationship criteria in Study 3.

Effect	Estimate	Error	df	t-value	Pr > |t|
Intercept	8.268	0.036	301	228.56	<0.001
Positive/Stable Composite (PSC)	0.253	0.032	1,180	7.99	<0.001
Enduring Concern Composite (ECC)	0.712	0.039	1,163	18.33	<0.001
Frequency of Interaction (Freq)	0.115	0.026	1,143	4.47	<0.001
PSC x ECC	−0.020	0.023	1,181	−0.85	0.394
PSC x Freq	−0.090	0.029	1,103	−3.14	0.002
ECC x Freq	−0.014	0.030	1,117	−0.47	0.636
PSC x ECC x Freq	0.006	0.013	1,115	0.45	0.655

#### Primary analyses

An objective of Study 3 was to examine the additive and interactive effects among the criteria in explaining relational belongingness, having reduced the seven standards to three factors. Relational belongingness was then regressed onto the main effect predictors and the interaction terms using the *SPSS Mixed Model* module to account for the data structure where relationships were nested within participants.

Strong evidence of additive effects was obtained as each of the three factors (Positive/Stable Interactions, Enduring Concern, and Frequency of Interaction) significantly predicted relational belongingness, *p*s < 0.001, with Pseudo $R^{2}$ s of 0.28, 0.50, and 0.08, respectively, when examined individually. The results indicate that frequency of interactions, positive/stable interactions, and enduring concern each contribute independently and additively to relational belongingness, with enduring concern accounting for most of the variance (i.e., for the 3 combined, Pseudo $R^{2}=.54$ ). In addition, one two-way interaction between frequency of interactions and positive/stability was obtained, *t* = −4.17, *p* < 0.001, but the effect is small and adds only 0.007 to the Pseudo $R^{2}$ for a total value of 0.547. A graph of the decomposed interaction is shown in Figure 8. As shown in the graph, belongingness decreased beyond the point explained by additive effects when both frequency of interactions and positive/stability were low. This interaction was not consistent with our construal of the strong form of the belongingness hypothesis in which we expected that belongingness would be especially high (i.e., over and above additive effects) when frequency of interactions, positive/stability, and enduring concern are all high. On the other hand, the interaction is nonetheless consistent with a weak form of the belongingness hypothesis. That is, belongingness is highest when all three factors are high but drops when frequency of interactions and positive/stability are low.

**Figure 8:**
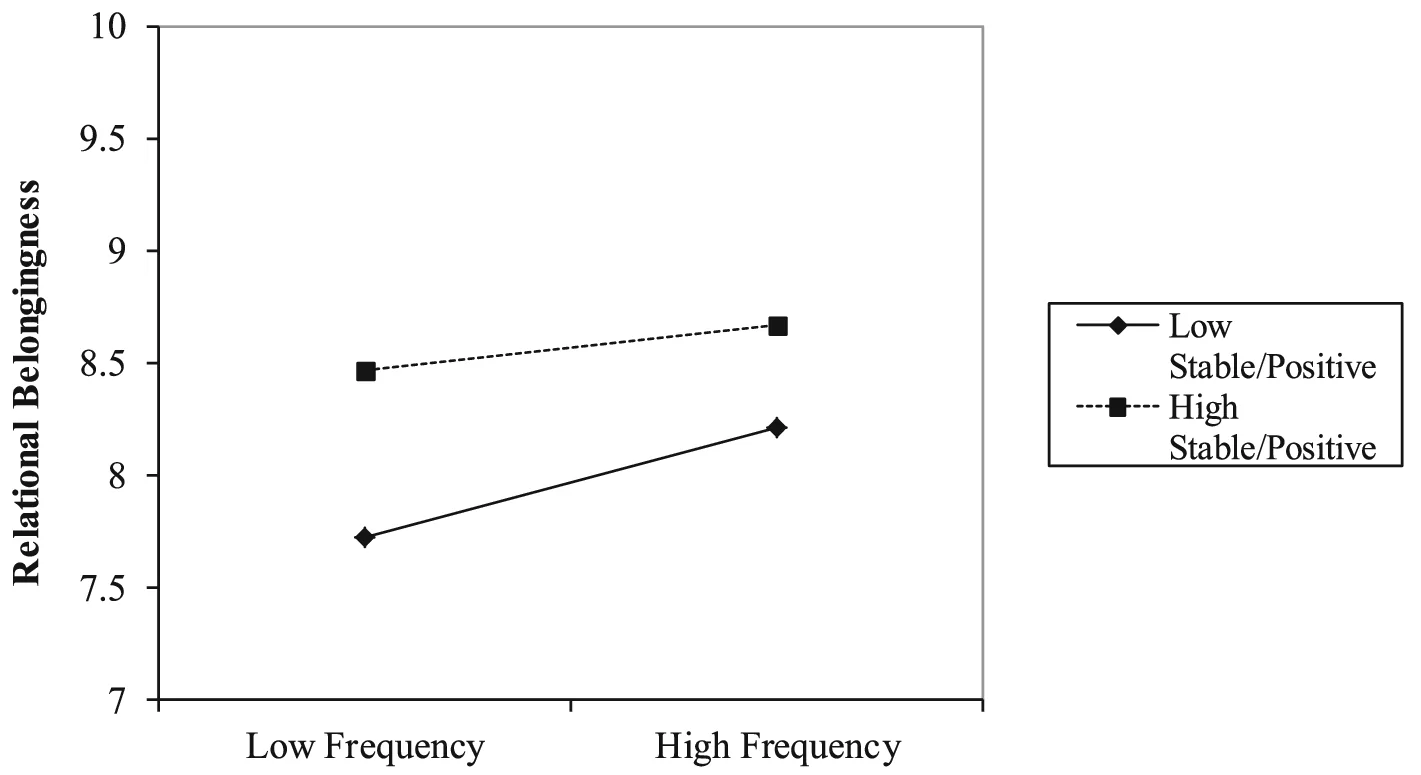
Study 3 display of interaction between positive/stable criteria and frequency criteria in predicting relational specific belongingness.

#### Supplemental analyses

The zero-order correlations between the variables used in the above analyses are included in Appendix C, Supplementary Analysis Table 4. All variables serving as criteria for whole relationships correlate significantly with relationship level belongingness. Interestingly, relationship level belonging correlates with person level numbers of whole, partial, and total numbers of relationships with r s of 0.24, −0.22, and 0.01, respectively, where the first are significant with p -values recalculated at the more conservative person level, p<.01 . These correlations suggest that the division of relationships into wholes vs. partial relationships has better predictive value than simply counting the total number of relationships. In addition, one particularly interesting finding is that frequency correlates negatively with total relationships, r=−.19,p<.01 Moreover, it is the only whole criterion variable that correlates negatively and significantly with total relationship count. One possible interpretation of this finding is that the greater the number of close relationships that one has, the less time one can spend with each person.

## Supplemental analyses across studies 1 to 3

The results of study 3 provide insight into the nature of whole relationships. We regard whole relationships as a form of high-quality relationships marked by primarily by positive/stable interactions and enduring concern. These features are like others that have been explored by various relationship researchers (e.g., Fletcher et al., 2000; Reis et al., 2004). What differentiates Baumeister and Leary (1995) conceptualization and our methodological operationalization from others is frequency of interactions. The multi-level analysis in Study 3 did not provide strong evidence for the frequency requirement. However, a limitation of the within-subject, multilevel approach is that ratings of belongingness can be influenced by people’s working models of what they expect each relationship should provide. Thus, we returned on a post-hoc basis to utilize the between subject features emphasized in the first two studies with which we began to construct an alternative test.

As noted earlier, Baumeister and Leary (1995) devoted two sections of their paper to partial relationships. These two conceptualizations can be used to probe systematic alterations of certain cutpoints we in a theoretically guided manner. For each of the three data sets, we compared how numbers of relationships correlate with belongingness and well-being for relationships that we classified as either (a) Whole (i.e., meeting all seven standard criteria used in this study that included frequency, positivity, and caring); (b) Type 1 Partial—infrequent interactions with a meaningful connection (i.e., we used the standard criteria for wholes except these relationships had interactions reported as occurring less than once a week); (c) Type II Partial—frequent interactions without a caring bond (i.e., we used the standard criteria for wholes except that participants reported that they neither agreed or strongly agreed that the individual cared for them or showed mutual concern.

Results are displayed in Table 3 (see also Supplemental Table 5 for differential correlations of total, whole, and partial relationships with belongingness and well-being). Across the three studies, the average correlation between numbers of close others and belongingness is 0.25 for whole relationships, but only 0.06 and −0.031 for Type I and Type II partial relationships. The same pattern holds with respect to well-being. These results support Baumeister and Leary (1995) propositions highlighting that high-quality (e.g., caring, positive, stable) relationships contribute to belonging primarily when combined with frequent interactions.

**Table 3:** Correlational relationships of whole relationships, Type I Partial (low frequency) relationships, and Type II Partial (low caring) relationships with belonging and well-being.

	T1 Partial: Low Freq, High Caring	T2 Partial: High Freq, Low Caring	Belonging	Well-being
Study 1 (*N* = 872)				
Whole as Specified	0.17**	0.02	0.25**	0.25**
Type 1 Partial: Low Freq		0.01	0.06	0.04
Type 2 Partial Low Caring			−0.03	−0.03
Study 2 (*N* = 581)				
Whole as Specified	0.11**	−0.03	0.25**	0.18**
Type 1 Partial: Low Freq		−0.04	0.01	0.02
Type 2 Partial: Low Caring			−0.05	−0.11**
Study 3 (*N* = 284)				
Whole as Specified	0.24**	−0.04	0.32**	0.32**
Type 1 Partial: Low Freq		−0.04	0.08	0.08
Type 2 Partial: Low Caring			−0.07	−0.06

**p < 0.01. All relationship type counts for this table are based on meeting criteria reported in Study 1 for positivity, low negativity, shared future, and stability. Whole relationships also meet the criteria for frequency, caring, and mutual concern. Type 1 Partial relationships meet all criteria except frequency. Type 2 Partial relationships meet all criteria except caring and mutual concern. Belonging and well-being are assessed as described in the Study 1 method section for Figures 3, 7.

## General discussion

The current findings provide support for specific features of the belongingness hypothesis that have been understudied since the Baumeister and Leary (1995) paper first appeared. First and foremost, fully satisfying (i.e., whole) relationships defined by the criteria set forth by Baumeister and Leary (1995) consistently showed positive associations with dimensions of perceived belongingness and subjective well-being. In addition, clear evidence was obtained showing that the pattern of associations involving whole relationships diverges from those involving partially satisfying (i.e., partial) relationships. Specifically, partial relationships were negatively (and most often significantly) associated with belongingness and well-being after controlling for a variety of other variables and effects in the model. As noted in Studies 2 and 3, these effects are not a simple function of the total number of relationships reported (see Appendix C Supplementary Analysis Table 5 for details across all 3 studies), and thus demonstrate it useful divide close relationships into those that meet Baumeister and Leary (1995) criteria—especially if counting relationships.

Perhaps most importantly, our research demonstrated that the criteria defined by the belongingness hypothesis for fully satisfying relationships can be applied to the evaluation of each close relationship reported by individuals. In this regard, the measurement scheme developed in this paper has implications for future research. By requiring that individuals evaluate each close relationship one at a time, it reduces reliance on generalized schemas that may influence ratings of relationship quality in alternative measures. The instrument for defining whole relationships is also noteworthy in that it includes items that are behavioral and objective in nature (e.g., frequency of interactions with others) and items that assess feelings and perceptions of meaningful connectedness (e.g., mutual affective concern). Moreover, not only is the instrument easy to administer, but the criteria are high in face validity, easily understood, specific, and brief, thereby making them a practical asset for professional intervention. Over time, the measurement scheme may prove useful to clinicians and the public at large.

### Quantity and quality: numbers of whole relationships

The current studies clarify the criteria for achieving fully satisfying relationships and address issues involving the desirable number of whole relationships that one seeks to form. Baumeister and Leary (1995) claimed that the satisfaction of forming additional relationships is diminished after individuals meet their minimum number of quality relationships, and the results from all three studies demonstrate a clear pattern of diminishing returns consistent with this proposition. Generally, participants who reported less than two to three whole relationships reported lower levels of perceived belongingness. However, there were progressively fewer benefits in adding whole relationships beyond some optimal number. The quadratic pattern was only statistically significant in 2 of 3 studies, and thus, future research will need to be done to determine if the effect is replicated in future research. All three studies find that individuals benefit from at least one whole relationship over none. Overall, reports of more than three to six whole relationships appear to provide fewer benefits compared to the first few. Given the diminishing benefits of numerous whole relationships found in 2 of the 3 studies, the findings raise speculative questions regarding the feasibility of maintaining more than 5 or 6 whole relationships—especially if frequent interactions are required and the relationships are not easily localized in one place such as a family home.

Our findings dovetail with those of Dunbar (1992, 2024). Research in this domain has shown evidence that humans tend to have layered friendship networks of 5 to 15 close relationships (i.e., 5 intimates and 15 close friends); the numbers then extend outward to approximately 50 good friends and 150 manageable social contacts. “Dunbar’s number” of 150 refers to the number of relationships that one could successfully track and manage as relatively stable social relationships, and research on social networks has yielded support for the theory and the layered number of contacts (Dunbar and Dunbar, 2010; Mac Carron et al., 2016; Zhou et al., 2005). The number stems from anthropological theory specifying that the best predictor of “human mean group size” could be based on the neocortex ratio measured against the rest of the brain—with a confidence interval spanning from approximately 100 to 230). Importantly though, Dunbar (2021) also emphasizes the importance of time limitations in managing close relationships. Relations require investments of time to form and maintain them, and time is a limited resource. This notion is consistent with our finding in Study 3 that frequency of interactions correlates negatively with the total number of relationships one reports. Since Dunbar (1992) claim, organizational structuring experts, developers of social networking software, and military strategists have endeavored to find optimal numbers in the search for efficiency, unit cohesion, and morale. Our findings add to evidence on the layered social networks put forth by Dunbar (2024) to indicate the optimal number of close, whole relationships among college students is likely somewhere in the range of five.

Research by Dunbar and others (e.g., Dunbar and Dunbar, 2010; Mac Carron et al., 2016) highlight themes consistent with factors that make for fully satisfying (i.e., whole) relationships via Baumeister and Leary (1995). These factors include mutual concern for one another, having a shared history, frequent contact, and trust. What previous research has not done, however, is to divide close relationships into those that fully meet a set of criteria vs. those that do not (e.g., whole vs. partial relationships). Our research shows that there is predictive utility in making this distinction, and makes the point that individuals need some number of high quality, fully satisfying, close or intimate relationships to fulfill their belongingness needs. To meet belongingness needs and ensure well-being in college students, our research suggests that the optimal number is somewhere in the range of 3 to 5 (+ or −2 on each side). However, such numbers might not work for other populations, and even among college students, and there may be other needs aside from those explored here requiring larger networks. Interestingly, our research demonstrates a clear trade-off of investing in partial relationships that might be defined by unreciprocated feelings of concern, infrequent interactions, or overly negative interactions. Our findings show that these partial relationships not only fail to support well-being but may detract from it.

Our post-hoc, supplemental analyses directly assessed two types of partial relationships that Baumeister and Leary (1995) emphasized. These analyses specifically show that college students need some whole relationships that include both frequency of contact and close bonds. Eliminating either of these criteria resulted in non-significant relations between each of those two types of partial relationships and belongingness. We think this is a better test of the Baumeister and Leary’s propositions than the test of the interaction conducted in Study 3 using multilevel modeling as that effect is influenced by participant’s expectations and beliefs about the value of each relationship. A college student who is asked if a parent contributes heavily to meeting their belongingness needs is likely to say yes if the relationship is warm and free of negativity—even if that parent lives 10 hours away. But that same college student may report lower than average belongingness when answering questions about belongingness and well-being that are not bound to specific relationships. The supplemental analysis we conducted across studies captures such phenomena on a between-persons basis relative free of expectancy effects; the methodology in Study 3 is unlikely to do so.

### Implications for managing relationships

This study may imply that individuals should consider strategically investing their time in a few healthy, whole relationships given evidence obtained concerning diminishing returns and the negative impact of maintaining partial relationships. According to Dunbar (2024), the key delimiting factor in managing all relationships is time. Most clearly, it requires time to have frequent contact with close others, and it takes quality time and energy to enjoy positive interactions, build empathy, and reciprocate caring. The greatest risk is for those individuals who lack a minimum number of fully satisfying relationships—yet attempt to convince themselves that what they have is sufficient, ultimately undermining their fundamental motivation to secure additional satisfying relationships that would subsequently enhance their levels of well-being. As suggested by Sirgy and Wu (2009), maintaining a proper balance in life may be the key to achieving subjective well-being. In the sphere of relationship maintenance, balance requires both nurturing at least a minimum quantity of interpersonal relationships and avoiding the risk of extending oneself past the point where one’s closest relationships become compromised.

This research may also have implications for the mediums through which individuals relate with one another to fulfill the criteria for fully satisfying relationships. We live in a society that is increasingly defined by electronic mechanisms of socializing. At this juncture it is still unclear whether one’s belongingness needs can be met through frequent telephone calls, online channels, or digital alternatives. Though adverse feelings manifested by distant close relationships may be alleviated by relationships with others in close physical proximity, imagine if *every* relationship was from afar. One may find it very difficult to feel adequate levels of belongingness and well-being without one or two close relationships with frequent interactions.

### Limitations and directions for future research

#### Causality

The present research demonstrates that having a few close relationships that meet the criteria stated in the original belongingness hypothesis (Baumeister and Leary, 1995) correlates systematically with well-being. As with most correlational research, however, we *cannot* make the case that meeting these criteria *causes* increased well-being. It is possible, for example, that those who are psychologically well-adjusted are more likely to develop relationships that meet the criteria advanced here. Future research might explore this reverse causality proposition as well as potential third variable propositions. Even if causality could be assumed, the current research cannot pinpoint the number of whole relationships required per person as a matter of individual differences. Indeed, there is clear evidence that there are individual differences in the need to belong or the drive to affiliate with others (Allen et al., 2021). Whether the minimum number of whole relationships needed for well-being is one, five or somewhere in between is likely to vary across individuals and such specification is outside the scope of our research.

#### Sample characteristics

Importantly, the present research was based on a collegiate population and cannot be generalized to other populations (e.g., children, middle-aged, or older adults). Cummings and Henry (1961) theorized that individuals naturally tend to disengage with larger social systems as they increase in age—and for older adults especially, it is easy to imagine that the total number of close relationships reported decreases. On the other hand, Carstensen (1992) and Tornstam (1997) proposed that even though there is a negative association between age and social interaction, it is a decrease in peripheral relationships that diminish, not close intimate relationships. Moreover, Shaw et al. (2007) demonstrated using longitudinal data social interactions with friends decreased with age, but no such decrease was evident in interactions with family members. Perhaps most importantly, college students live both in milieu with numerous social contacts, and, critically, are in a prime developmental period for developing intimate relationships (Arnett, 2000))—potentially making them prone to depression and lack of well-being if lacking at least one or two whole relationships. Clearly, future research is needed to examine age-related differences in how individuals report levels of fully and partially satisfying relationships.

#### Instrument items and cutpoints

Future research may also be needed to either verify or refine the measures and cutoff values used here to define whole relationships. For our research, we established cutpoints that were specific to a sample of college students. For example, to assess frequency of interaction we used a cutpoint of, “*at least once per week*.” It is possible that “once per week” might not be an adequate cutoff value for older populations busy with work, school, and children. In addition, assessing perceptions of how a relationship continues into the foreseeable future differs dramatically depending upon whether the relationship is familial or social. For example, a default response for a familial relationship would often be, “*all of my life.*” Yet, that same individual may have a current friendship that better meets their belongingness needs but not respond accordingly because of lessor expectations for continuance of such relationships into the foreseeable future. Further refinement of the belongingness criteria will require a more diverse sample representing the broader population to adequately assess these forms of variability.

Further refinement of the belongingness criteria may also benefit from the use of approaches that include Item Response Theory or machine learning for selecting cutpoints. It is important to note that the current paper was not designed to provide a definite set of criteria and cutpoints, but rather to test the proposition that whole relationships—per the criteria laid out by Baumeister and Leary (1995)—differ in their relation to sense of belonging and well-being than do partially satisfying relationships in a population of college students. Having now derived an initial framework for this empirical test among college students, future research will be needed to refine the instrument and examine whether these cutpoints will operate similarly among other populations who are middle-aged or older.

Such follow-up studies may involve using multiple items to assess a more limited set of criteria as informed by Study 3. Here we found evidence that the 7 initial criteria are reduced to 3 dimensions, with enduring concern (i.e., mutual concern embedded in a relationship expected to last) accounting for the greatest amount of variance in perceptions of how each particular relationship makes them feel that they belong (which is not necessarily equivalent to general belongingness). In any case, derivations of the criteria used in this study need to be short for both research purposes and individual use as the instrument needs to be applied to each individual relationship evaluated. As for individual or clinical use, it is also important that those using the instrument avoid reifying the cutpoints. There will certainly be some measurement error surrounding each cutpoint used, and these may vary based on the type of relationship evaluated as well as individual response tendencies. Again, our usage of these cutpoints served the research imperative of testing propositions of the belongingness hypothesis.

Further analyses will also be required to examine the specific contributions of each of the seven criteria. For instance, Baumeister and Leary (1995) were tentative in their conclusion regarding the role of mutual affective concern in defining whole relationships. Our analyses indicate that there is substantial variability in participants’ reports of mutual affective concern. Thus, mutual affective concern appears useful in differentiating whole relationships from partials. This finding is consistent with equity theory which posits that people are generally most satisfied with relationships when they perceive that the ratio of their inputs to outputs matches that of their relationship partner (e.g., Adams and Freedman, 1976; Davidson, 1984). Further research exploring the contributions of each belongingness criterion is needed, but it is important to recognize the difficulties inherent in this task. That is, the criteria are used to evaluate relationships reported within person, and then these data are aggregated and used to evaluate overall belongingness at the level of the person.

#### Implications for social networks

Future research should also explore how relationships are embedded in social networks. A key element of social networks that relates to time to manage relationships concerns network density. If relationships are embedded in a family or group that facilitate spending time with multiple individuals at once, then the resources to maintain numerous relationships may be manageable. Moreover, the group may confer benefits for belongingness unavailable in other relationships. For instance, a relationship that is lost from a dense network may be—to some degree—buffer the adverse impact of that loss via others in the group or family who share the same common history together (Jetten et al., 2012). As social networks become more diffuse, it would seem more difficult to find the time to manage and nurture more than a few intimate or close relationships—though the work of Jetten et al. (2012) highlight the social advantages of multiple group memberships. These ideas are speculative but deserve further attention.

Future research should also explore how whole and partial relationships function to meet belongingness needs in the context of other influences on belongingness. In their review of the literature, Hirsch and Clark (2019) find evidence of multiple paths that explain belongingness—noting that communal relationships (or close relationships as studied here) is only one such path. They argue that other pathways center on social status (or feeling of value), group memberships, and minor social interactions. Hirsh and Clark argue that these may function in either additive ways or substitute for one another. College students, for instance, tend to live in environments that provide for a multitude of minor social interactions. If these are considered, it is possible that fewer whole relationships are needed. On the other hand, it is also possible that older adults who do not live in such an environment have even a greater need for a few whole relationships. Future research is needed to explore these issues.

## Final message

The Baumeister and Leary (1995) review is arguably the premier article responsible for launching a wave of research involving belongingness. In this research, we examined principal components of the theory not previously addressed and found support the key proposition that college students benefit from relationships that fulfill a set of criteria to quality as fully satisfying relationships. Moreover, these relationships do not function in the same manner as partially satisfying relationships. However, there remain numerous ideas and propositions stemming from their theoretical work that still require in-depth examination. Despite interventions in place for guiding relationship modification (e.g., marriage counseling), what is missing in the literature, education, and practice is a set of empirically tested guidelines that provide for simple evaluations of relationships and goal setting. In this paper we have provided a brief distillation of the belongingness criteria that can be easily taught, understood, and utilized for constructing new relationship goals and charting one’s progress toward fulfilling one of the most basic of human needs.

## Data Availability

The datasets presented in this study can be found in online repositories. The names of the repository/repositories and accession number(s) can be found at: https://osf.io/hecqd.
