# Supplementary material for: A direct test of the belongingness hypothesis: how fully vs. partially satisfying relationships relate to perceived belongingness and well-being
Source: Front Psychol. 2026 Oct 7;17:1830244. doi: 10.3389/fpsyg.2026.1830244 (PMC13685428; doi:10.3389/fpsyg.2026.1830244)
Supplement: Supplementary file 1 [file Supplementary_file_1.DOCX]

**Supplemental Appendix A**

How frequently do you interact with this person?

1. **Everyday**
2. **Almost every day**
3. **Few times a week**
4. ***Once a week***
5. Few times a month
6. Once a month
7. Several times a year
8. About once a year
9. Every few years
10. Almost never
11. Never

How long do you feel that your relationship with this person will continue into the future?

1. **All my life**
2. ***10 to 20 years***
3. 5 to 10 years
4. 1 to 5 years
5. 6 months to 1 year
6. 1 to 6 months
7. Less than 6 months

Over the course of time, my relationship with this person has been stable.

1. **Strongly agree**
2. ***Agree***
3. Somewhat agree
4. Somewhat disagree
5. Disagree
6. Strongly disagree

My interactions with this person are negative and conflicting.

1. Always
2. Almost always
3. Often
4. ***Sometimes***
5. **Seldom**
6. **Almost Never**
7. **Never**

My interactions with this person are positive.

1. **Always**
2. **Almost always**
3. ***Often***
4. Sometimes
5. Seldom
6. Almost Never
7. Never

I feel that this person is concerned for my well-being.

1. ***Strongly agree***
2. ***Agree***
3. Somewhat agree
4. Somewhat disagree
5. Disagree
6. Strongly disagree

I feel that this person has as much concern for me as I have for them.

1. ***Strongly agree***
2. ***Agree***
3. Somewhat agree
4. Somewhat disagree
5. Disagree
6. Strongly disagree

*Note.* Reponses in bold were acceptable responses for defining fully satisfying (i.e., whole) relationships. Responses in bold italics represent the cutoff values. Any response for any of the items that fell below the cutpoint resulted in a relationship being recorded as a partially satisfying relationship.

**Supplemental Appendix B**

**General Belongingness.** The General Belongingness Scale (GBS; Malone et al., 2012)

was rated on a 7-point scale ranging from 1 (*strongly disagree*) to 7 (*strongly agree*). In Study 2 and Study 3, the item, “I fit in with my friends and family” was changed to, “Friends and family do not involve me in their plans,” and the item, “I feel like a social outcast” was changed to, “I have a sense of belonging.” The final version of the GBS uses the items from Studies 2 and 3.

***Study 1 Items***

1. I feel connected with others.
2. I fit in with my friends and family.
3. When I am with other people, I feel like a stranger. (reverse-scored)
4. Because I don’t belong, I feel distant during the holiday season. (reverse-scored)
5. I have a place at the table with others.
6. I have close bonds with family and friends.
7. I feel isolated from the rest of the world. (reverse-scored)
8. I feel like a social outcast. (reverse-scored)
9. When I am with other people, I feel included.
10. I feel accepted by others.
11. I feel as if people don’t care about me. (reverse-scored)
12. I feel like an outsider. (reverse-scored)

***Study 2 and Study 3 Items***

1. When I am with other people, I feel included.
2. I have close bonds with family and friends.
3. I feel like an outsider. (reverse-scored)
4. I feel as if people don't care about me. (reverse-scored)
5. I feel accepted by others.
6. Because I don't belong, I feel distant during the holiday season. (reverse-scored)
7. I feel isolated from the rest of the world. (reverse-scored)
8. I have a sense of belonging.
9. When I am with other people, I feel like a stranger. (reverse-scored)
10. I have a place at the table with others.
11. I feel connected with others.
12. Friends and family do not involve me in their plans. (reverse-scored)

**Supplemental Appendix C**

**Supplemental Table 1**

*Correlations and Descriptive Statistics for Study 1*

|  | 1. | 2. | 3. | 4. | 5. | 6. | 7. | 8. |
| --- | --- | --- | --- | --- | --- | --- | --- | --- |
| 1. Whole Relationships | --- |  |  |  |  |  |  |  |
| 2. Partial Relationships | .041 | --- |  |  |  |  |  |  |
| 3. General Belonging | .247^***^ | -.078^*^ | --- |  |  |  |  |  |
| 4. Sense of Belonging | .225^***^ | -.051 | .860^***^ | --- |  |  |  |  |
| 5. Loneliness | -.237^***^ | .122^***^ | -.797^***^ | -.763^***^ | --- |  |  |  |
| 6. Life Satisfaction | .220^***^ | -.102^**^ | .585^***^ | .555^***^ | -.563^***^ | --- |  |  |
| 7. Happiness | .207^***^ | -.134^***^ | .670^***^ | .616^***^ | -.633^***^ | .650^***^ | --- |  |
| 8. Depression | -.215^***^ | .086^*^ | -.634^***^ | -.648^***^ | .590^***^ | -.551^***^ | -.596^***^ | --- |
|  |  |  |  |  |  |  |  |  |
| Means | 2.42 | 1.79 | 5.80 | 3.33 | 1.94 | 4.81 | 5.17 | 0.77 |
| SDs | 2.05 | 1.88 | 1.17 | 0.64 | 0.62 | 1.46 | 1.27 | 0.53 |

*Note.* **p* < .05. ***p* < .01. ****p* < .001.

**Supplemental Table 2**

*Correlations and Descriptive Statistics for Study 2*

|  | 1. | 2. | 3. | 4. | 5. | 6. | 7. | 8. |
| --- | --- | --- | --- | --- | --- | --- | --- | --- |
| 1. Whole Relationships | --- |  |  |  |  |  |  |  |
| 2. Partial Relationships | -.043 | --- |  |  |  |  |  |  |
| 3. General Belonging | .243^***^ | -.171^***^ | --- |  |  |  |  |  |
| 4. Sense of Belonging | .220^***^ | -.125^**^ | .653^***^ | --- |  |  |  |  |
| 5. Loneliness | -.218^***^ | .164^***^ | -.731^***^ | -.668^***^ | --- |  |  |  |
| 6. Life Satisfaction | .169^***^ | -.123^**^ | .501^***^ | .458^***^ | -.496^***^ | --- |  |  |
| 7. Happiness | .156^***^ | -.155^***^ | .592^***^ | .590^***^ | -.617^***^ | .673^***^ | --- |  |
| 8. Depression | -.196^***^ | .111^**^ | -.496^***^ | -.625^***^ | .551^***^ | -.459^***^ | -.569^***^ | --- |
|  |  |  |  |  |  |  |  |  |
| Means | 2.18 | 1.42 | 5.73 | 3.25 | 1.91 | 4.84 | 5.24 | 0.77 |
| SDs | 1.84 | 1.72 | 0.98 | 0.66 | 0.61 | 1.33 | 1.26 | 0.51 |

*Note.* **p* < .05. ***p* < .01. ****p* < .001.

**Supplemental Table 3**

*Correlations and Descriptive Statistics for Study 3*

|  | 1. | 2. | 3. | 4. | 5. | 6. | 7. | 8. |
| --- | --- | --- | --- | --- | --- | --- | --- | --- |
| 1. Whole Relationships | --- |  |  |  |  |  |  |  |
| 2. Partial Relationships | .022 | --- |  |  |  |  |  |  |
| 3. General Belonging | .287^***^ | -.089 | --- |  |  |  |  |  |
| 4. Sense of Belonging | .295^***^ | -.057 | .767^***^ | --- |  |  |  |  |
| 5. Loneliness | -.308^***^ | .020 | -.702^***^ | -.756^***^ | --- |  |  |  |
| 6. Life Satisfaction | .298^***^ | -.025 | .524^***^ | .537^***^ | -.534^***^ | --- |  |  |
| 7. Happiness | .299^***^ | -.066 | .630^***^ | .657^***^ | -.643^***^ | .596^***^ | --- |  |
| 8. Depression | -.209^***^ | .030 | -.563^***^ | -.672^***^ | .627^***^ | -.504^***^ | -.589^***^ | --- |
|  |  |  |  |  |  |  |  |  |
| Means | 2.42 | 1.82 | 5.61 | 3.31 | 1.98 | 4.89 | 5.25 | 0.75 |
| SDs | 2.13 | 2.00 | 1.02 | 0.59 | 0.66 | 1.30 | 1.20 | 0.47 |

*Note.* **p* < .05. ***p* < .01. ****p* < .001.

**Supplemental Table 4**

*Correlations Between Whole, Partial, and Total Relationship and the Nine Criteria for Study 3*

|  | 1. | 2. | 3. | 4. | 5. | 6. | 7. | 8. | 9. | 10. | 11. |
| --- | --- | --- | --- | --- | --- | --- | --- | --- | --- | --- | --- |
| 1. Frequency | --- |  |  |  |  |  |  |  |  |  |  |
| 2. Positive | .061^*^ | --- |  |  |  |  |  |  |  |  |  |
| 3. Negative | -.059^*^ | .578^***^ | --- |  |  |  |  |  |  |  |  |
| 4. Stable | .203^***^ | .587^***^ | .385^***^ | --- |  |  |  |  |  |  |  |
| 5. Forever | .049 | .173^***^ | .057^*^ | .276^***^ | --- |  |  |  |  |  |  |
| 6. Caring | .113^***^ | .295^***^ | .174^***^ | .429^***^ | .357^***^ | --- |  |  |  |  |  |
| 7. Concern | .217^***^ | .241^***^ | .110^***^ | .369^***^ | .353^***^ | .530^***^ | --- |  |  |  |  |
| 8. Belonging | .280^***^ | .472^***^ | .288^***^ | .575^***^ | .315^***^ | .569^***^ | .562^***^ | --- |  |  |  |
| 9. Wholes | .039 | .244^***^ | .221^***^ | .268^**^ | .157^***^ | .293^***^ | .202^***^ | .238^***^ | --- |  |  |
| 10. Partials | -.313^***^ | -.130^***^ | -.036 | -.243^***^ | -.185^***^ | -.261^***^ | -.179^***^ | -.223^***^ | -.009 | --- |  |
| 11. Total Relations | -.194^***^ | .080^**^ | .131^***^ | .017 | -.018 | .023 | .017 | .011 | .704^***^ | .704^***^ | --- |

*Note.* **p* < .05. ***p* < .01. ****p* < .001. Whole, Partials, and Total Relationships are assessed at the between group level and the rest of the variables are assessed at the within group level.

**Supplemental Table 5**

*Correlations Between Whole Relationships, Partial Relationships, Belonging, and Well-being*

|  | Wholes | Partials | Belonging | Well-being |
| --- | --- | --- | --- | --- |
| Study 1 (*N* = 872) |  |  |  |  |
| Total Relations | .732** | .710** | .122** | .085* |
| Wholes |  | .041 | .253** | .246** |
| Partials |  |  | -.082* | -.130** |
| Study 2 (*N* = 581) |  |  |  |  |
| Total Relations | .716** | .667** | .067 | .024 |
| Wholes |  | -.043 | .254** | .182** |
| Partials |  |  | -.176** | -.159** |
| Study 3 (*N* = 284) |  |  |  |  |
| Total Relations | .737** | .683** | .187** | .194** |
| Wholes |  | .009 | .323** | .324** |
| Partials |  |  | -.073 | -.063 |

*Note.* **p* < .05. ***p* < .01. Belonging and Well-being are assessed here as factor scores representing the variables displayed in the major analytic model shown in Figure 1.
